# The dual functions of the GTPase BipA in ribosome assembly and surface structure biogenesis in *Salmonella enterica* serovar Typhimurium

**DOI:** 10.1371/journal.ppat.1013047

**Published:** 2025-04-09

**Authors:** Eunsil Choi, Eunwoo Ryu, Donghwee Kim, Ji-Won Byun, Kahyun Kim, Minho Lee, Jihwan Hwang

**Affiliations:** 1 Department of Microbiology, Pusan National University, Busan, Republic of Korea; 2 Microbiological Resource Research Institute, Pusan National University, Busan, Republic of Korea; 3 Department of Microbiology, College of Medicine, Hallym University, Chuncheon, Republic of Korea; 4 Institute of Medical Science, College of Medicine, Hallym University, Chuncheon, Republic of Korea; University of Tübingen, GERMANY

## Abstract

The bactericidal/permeability-increasing protein (BPI)-inducible protein A (BipA) is a highly conserved protein in Gram-negative bacteria that is structurally similar to translational GTPases such as IF2, EF-Tu, and EF-G. Our previous research showed that deleting *bipA* in *Escherichia coli* at 20°C leads to a defect in 50S ribosomal assembly and impaired lipopolysaccharide (LPS) synthesis. This LPS defect activates the Regulator of Capsule Synthesis (Rcs) pathway, resulting in an overproduction of capsular polysaccharides, a reduction in biofilm formation, and decreased flagella-mediated motility. In this study, we aimed to elucidate the role of BipA in the pathogenicity of *Salmonella enterica* serovar Typhimurium. We constructed *bipA* deletion mutants in two pathogenic *S*. Typhimurium strains, SL1344 and 14028, as well as in the attenuated strain LT2. Our ribosome profiling experiments using the mutant *S*. Typhimurium strains revealed a defect in ribosome assembly at 20°C, with the accumulation of abnormal 50S ribosomal subunits. We further demonstrated that the absence of BipA in *S*. Typhimurium impaired LPS biosynthesis at 20°C, compromising membrane integrity and presumably activating the Rcs pathway. This activation altered virulence factors, including reduced biofilm formation, particularly in the 14028Δ*bipA* strain. Furthermore, the SL1344Δ*bipA* and 14028Δ*bipA* strains exhibited significantly decreased swimming motility at 20°C compared to 37°C, confirmed by microscopic observation showing fewer flagella at 20°C. Subsequently, both strains exhibited a significant reduction in invasion capability and cytotoxicity toward human intestinal epithelial cells (HCT116). This functional attenuation was corroborated by the decrease in virulence observed in the 14028Δ*bipA* strain in a mouse model. Our findings suggest that, in *S*. Typhimurium, BipA functions as a bacterial fitness factor, contributing to ribosome assembly, LPS synthesis, and virulence-related processes, particularly under stress conditions relevant to host environments.

## Author summary

Pathogenic bacteria, such as *Salmonella enterica* serovar Typhimurium, utilize specialized proteins and mechanisms to facilitate infection and evade the host’s immune response. A critical aspect of these mechanisms involves modifying the cell envelope structures to protect the bacteria from host defenses. Understanding these processes is essential for developing new strategies to combat bacterial infections. Our study examines the role of the BipA protein in *S*. Typhimurium, which is crucial for ribosome assembly and the formation of surface structures, such as lipopolysaccharide, biofilm, and flagella. These structures are essential for bacterial motility, survival under stresses, and pathogenicity. We found that deleting *bipA* disrupted ribosome assembly at low temperatures. BipA is pivotal for lipopolysaccharide and flagella production under stressed conditions and is necessary for bacterial movement and host cell invasion. These findings highlight the significance of BipA in bacterial adaptation to stress and its regulatory role in key virulence determinant. Our study offers new insights into bacterial survival strategies and identifies potential targets for antimicrobial treatment development.

## Introduction

Bacterial GTPases play crucial regulatory roles in various cellular processes, such as protein synthesis and adaptation to environmental stress [1,2]. Among these, bactericidal/permeability-increasing protein (BPI)-inducible protein A (BipA), also known as Tyrosine phosphorylation protein A (TypA), is a member of the translational GTPase family [3], and its structure closely resembles those of EF-G and LepA within this group [4]. BipA is upregulated in *Salmonella enterica* serovar Typhimurium when exposed to the BPI antimicrobial protein [5], which is released by innate immune phagocytic cells, particularly neutrophils [6–8].

BPI is a ~55 kDa single-chain protein, first identified in the primary granules of human neutrophils, known for its antimicrobial efficacy and high selectivity toward Gram-negative bacteria [9]. BPI consists of a highly cationic N-terminal domain that shares a 45% sequence identity with lipopolysaccharide (LPS)-binding protein and an acidic C-terminal domain [10–12]. The cationic N-terminal domain of BPI has a strong affinity for the lipid A-inner core region of LPS [13]. LPS, a key component of the outer membrane of Gram-negative bacteria, is stabilized by divalent ions, such as calcium and magnesium [14,15]. When BPI binds to lipid A, it displaces these ions [16], leading to outer membrane destabilization, increased permeability [17], and disruption of cell division [18]. While the N-terminal domain exhibits antibiotic activity, the C-terminal domain promotes opsonophagocytosis by directing BPI-coated bacteria to neutrophils [19].

In response to BPI cytotoxicity, bacteria trigger the expression of various genes [20]. A well-known strategy for counteracting BPI involves activating genes responsible for modifying or repairing the LPS structure [21]. Research has shown that increasing the length of the polysaccharide chain in LPS enhances resistance to BPI in *E. coli*, *S.* Typhimurium, and *Proteus mirabilias* [22–24]. This elongation may hinder the access of BPI to the lipid A-inner core region. Consequently, the expression of *bipA* upon BPI exposure may serve as a mechanism for *Salmonella* to respond to BPI by orchestrating structural changes in LPS. This phenomenon has been observed in *bipA*-deleted *Yersinia pestis* and enteropathogenic *E. coli*, both of which exhibit increased sensitivity to BPI [25,26].

Furthermore, our previous study revealed that *E. coli* BipA is promptly induced at low temperatures in a cAMP-CRP-dependent manner [27]. Deleting *bipA* in *E. coli* K-12 led to a growth defect at low temperatures due to the loss of BipA binding to the 50S ribosomal subunit, leading to a defect in ribosome assembly. Additionally, we observed defects in LPS biosynthesis, which activated the Rcs signaling pathway at low temperatures. This activation enhanced capsule production, inhibited biofilm formation, and decreased flagella-mediated motility [28]. Similarly, *bipA* deletion in *Pseudomonas aeruginosa* resulted in reduced biofilm formation and increased antibiotic susceptibility [29]. In *Y. pestis*, BipA enhances virulence and resistance to neutrophil attacks [26]. Similarly, in *P. aeruginosa*, *bipA* deletion weakens biofilm formation and downregulates the type III secretion system (T3SS), reducing bacterial uptake by human macrophages [29]. These findings establish BipA as a crucial regulator of temperature-dependent colony morphology, motility, and pathogenicity associated with surface structures.

In this study, we comprehensively investigated the diverse roles of BipA in ribosome assembly, stress adaptation, virulence-associated functions, and pathogenesis in two pathogenic *S*. Typhimurium strains, SL1344 and 14028, as well as in the attenuated pathogenic strain LT2, by deleting the *bipA* gene in each strain. Our data indicate that the role of *Salmonella enterica* BipA in 50S ribosome biogenesis is temperature-dependent, with *bipA* mutant strains showing defects in surface structures, such as LPS, biofilm, flagella, and T3SS at low temperatures. Conversely, at 37°C, reduced ATP levels and increased sensitivity to reactive oxygen species (ROS) likely diminish the pathogenicity of the SL1344 and 14028 strains. Our findings provide insights into BPI resistance through BipA-mediated changes in LPS and highlight BipA as a key fitness factor at low temperatures, facilitating adaptation to stress conditions, such as cold, ROS, and exposure to antimicrobial effectors.

## Materials and methods

### Ethics statement

All animal study procedures were approved by the Institutional Animal Care and Use Committee of Hallym University (Hallym 2024-2).

### Bacterial strains and growth conditions

All bacterial strains used in this study are listed in Table 1. The *E. coli* and *S*. Typhimurium strains were cultured at either 37°C or 20°C in Luria–Bertani (LB) medium supplemented with ampicillin (Amp, 100 μg/mL), chloramphenicol (Cm, 50 μg/mL), or kanamycin (Kan, 50 μg/mL) as required. To delete the *bipA* gene in the *Salmonella* strains, the *SebipA*::kan cassette was amplified through PCR using pKD13 as a template. The primers are listed in S1 Table. This cassette was introduced into the SL1344, 14028, and LT2 *S*. Typhimurium strains—which contained the pKD46 plasmid—through electroporation, following the methodology described by Datsenko and Wanner [30]. After transformation, kanamycin-resistant cells were confirmed through PCR to verify gene deletion. To delete *rcsA* and *lon*, MG1655 and ESC26 (Δ*bipA*) cells were subjected to P1 transduction using lysates from JW1935 (*rcsA*::kan) or JW0429 (*lon*::kan) cells, resulting in the MG1655Δ*rcsA*, MG1655Δ*bipA*Δ*rcsA*, and MG1655Δ*lon* strains. For growth monitoring, the optical density at 600 nm (OD_600_) was measured using a BioTek Epoch2 microplate spectrophotometer (Agilent Technologies).

**Table 1:** Bacterial strains and plasmids.

Strains	Description	References
MG1655	F^−^ λ^−^ *ilvG*- *rfb*-50 *rph*-1, *E. coli* K-12	[31]
MG1655Δ*bipA*	*bipA*::kan, MG1655 (ESC19)	[27]
MG1655Δ*rcsF*	*rcsF*::kan, MG1655 (ESC53)	[28]
MG1655Δ*bipA*Δ*rcsF*	Δ*bipA, rcsF*::kan, MG1655 (ESC54)	[28]
MG1655Δ*rcsA*	*rcsA*::kan, MG1655	This study
MG1655Δ*bipA*Δ*rcsA*	Δ*bipA, rcsA*::kan, MG1655	This study
MG1655Δ*lon*	*lon*::kan, MG1655	This study
JW0429	*lon::kan*, BW25113	[32]
JW1935	*rcsA*::kan, BW25113	[32]
JW3595	*waaF*::kan, BW25113	[32]
JW3606	*waaG*::kan, BW25113	[32]
JW3607	*waaQ*::kan, BW25113	[32]
SL1344	wild-type, *S*. Typhimurium (High virulence strain)	[33]
SL1344Δ*bipA*	*bipA*::kan, *S*. Typhimurium SL1344	This study
14028	wild-type, *S*. Typhimurium (High virulence strain)	ATCC
14028Δ*bipA*	*bipA*::kan, *S*. Typhimurium 14028	This study
LT2	wild-type, *S*. Typhimurium (Laboratory strain)	[34]
LT2Δ*bipA*	*bipA*::kan, *S*. Typhimurium LT2	This study
BL21(DE3)	F^−^ *ompT hsdS*_B_ (r_B_^−^ m_B_^−^) *gal dcm* (DE3), *E. coli*	Novagen
**Plasmids**		
pKD13	FRT-kan-FRT, Amp^R^, Kan^R^	[30]
pKD46	P_araB_, γ β exo, Rep^ts^, Amp^R^	[30]
pET28a	T7 promoter, N-terminal His_6_-tag, Kan^R^	Novagen
pET28BipA	*E. coli bipA*^+^, pET28a	[27]
pET28BipA_N128D_	*E. coli bipA*_N128D_, pET28a	[27]
pET28SeBipA	*S*. Typhimurium *bipA*^+^, pET28a	This study
pET28SeBipA_N128D_	*S*. Typhimurium *bipA*_N128D_, pET28a	This study
pACYC177	*ori* p15A, Amp^R^, Kan^R^	New England Biolabs
pACYC177BipA	*E. coli bipA*^+^, pACYC177	This study
pACYC177BipA_N128D_	*E. coli bipA*_N128D_, pACYC177	This study
pACYC177SeBipA	*S*. Typhimurium *bipA*^+^, pACYC177	This study
pACYC177SeBipA_N128D_	*S*. Typhimurium *bipA*_N128D_, pACYC177	This study
pACYC184	*ori* p15A, Cm^R^, Tc^R^	New England Biolabs
pACYC184PagP	*E. coli pagP*^+^, pACYC184	This study
pIN-A	*lpp*_p_, *lac*_po_, Amp^R^, expression vector	[35]
pINFliC	*E. coli fliC*, pIN-A	This study
pINSeFliC	*S*. Typhimurium *fliC*, pIN-A	This study
pINSeFljB	*S*. Typhimurium *fljB*, pIN-A	This study

Amp^R^; Ampicillin resistance, Kan^R^; Kanamycin resistance, Cm^R^; Chloramphenicol resistance, Tc^R^; Tetracycline resistance.

### Plasmid construction

To construct pACYC177BipA, pACYC177SeBipA, and pACYC184PagP, DNA fragments containing the open reading frame and its flanking regions were obtained through PCR using the *E. coli* and *S.* Typhimurium genomes, with the primers listed in S1 Table. The PCR-generated DNA fragments were ligated into the pACYC177 vector at the *Psi*I site or into the pACYC184 vector at the *Eco*RV site. Using these clones and mutant primers, site-directed mutagenesis was conducted to generate pACYC177BipA_N128D_ and pACYC177SeBipA_N128D_. To construct the overexpression vectors pET28SeBipA and pET28SeBipA_N128D_, the DNA fragments amplified from pACYC177SeBipA and pACYC177SeBipA_N128D_ were digested with the restriction enzymes *Nde*I and *Hin*dIII and then inserted into the corresponding sites of the expression vector pET28a. Similarly, the *fliCs* and *fljB* DNA fragments were amplified using *E. coli* or *S.* Typhimurium genomes as templates, with the primer sets listed in S1 Table. These fragments were subsequently digested with *Nde*I and *Hin*dIII and ligated into the corresponding sites of the pIN-A vector, resulting in the construction of pINFliC, pINSeFliC, and pINSeFljB. All plasmids utilized in this study are listed in Table 1.

### Quantitative real-time PCR

Total RNA was extracted from 6 × 10^8^ cells using the hot phenol method, as previously described [36]. Fifty micrograms of purified RNAs were incubated with RNase-free DNase I (TaKaRa Bio) at 37°C for 30 min. After DNase I removal through phenol extraction and ethanol precipitation, RNA quality was assessed using agarose gel electrophoresis, and concentration was measured with the NanoDrop One Spectrophotometer (Thermo Fisher Scientific). For cDNA synthesis, 1 μg of total RNA was mixed with 20 pmol of gene-specific primer in a total volume of 14 μL and heated to 65°C for 5 min. After cooling at 4°C for 2 min, a reaction buffer, dithiothreitol, and Moloney murine leukemia virus RNase H–RTase (BioFact RT-Kit) were added. The reverse transcription reaction was conducted at 50°C for 30 min and then inactivated at 95°C for 5 min. A quantitative real-time polymerase chain reaction (qRT-PCR) was performed using a QuantStudio 3 Real-Time PCR Instrument (Applied Biosystems), as previously described [27]. The 16S rRNA gene served as an endogenous control to normalize the expression levels of *bipA*, *cspA*, *gmd*, *fimD*, flagella-related genes, and *Salmonella* pathogenicity island 1 (SPI-1) genes associated with the T3SS. Relative quantification values were calculated using the comparative CT method [37].

### Western blot

After SDS-PAGE, the gel was immersed in transfer buffer (20% methanol in 1X SDS running buffer) and agitated for 20 min. The proteins were then transferred to a PVDF membrane (GE Healthcare) at 10 V for 30 min using a semidry transfer system. The membrane was then immersed in 5% skim milk in Tween-Tris-buffered saline (TTBS) and blocked for 3 h at room temperature. Primary antibodies, including anti-BipA, anti-CspA, anti-OmpA, and anti-FliC, were diluted to ratios ranging from 1:5,000 to 1:20,000 in 3% skim milk in TTBS and applied to the membrane. After overnight incubation with the antibodies at 4°C, the membrane was washed with TTBS and incubated with alkaline phosphatase–conjugated antirabbit antibody (1:10,000) in 3% skim milk in TTBS at 4°C for 1 h. Protein detection was conducted using 5-bromo-4-chloro-3-indolyl-phosphate and nitro blue tetrazolium.

### Protein overexpression and purification

*E. coli* BL21(DE3) cells were transformed with a pET28a-derived vector and cultured at 37°C until the OD_600_ reached 0.5–0.6. To induce protein expression, isopropyl β-D-thiogalactopyranoside was added to a final concentration of 0.5 mM, and the cultures were incubated at 15°C overnight. The induced cells were harvested by centrifugation at 4°C, and the cell pellets were washed with 10 mM Tris-HCl (pH 6.8), followed by another round of centrifugation. For the purification of wild-type and mutant His_6_-tagged BipA proteins, the cell pellets were resuspended in 20 mL of Buffer A (20 mM Tris-HCl [pH 7.5], 300 mM NaCl, and 2 mM β-mercaptoethanol [BME]), lysed by sonication, and centrifuged at 10,000 × g at 4°C for 25 min to remove the insoluble fraction. The supernatant was then ultracentrifugated at 70,000 × g at 4°C for 1 h using a Beckman 70Ti rotor to remove the membrane fraction. The soluble fractions were applied to a Ni-NTA agarose resin (Qiagen) column pre-equilibrated with Buffer A. The column was washed with 40 column volumes of Buffer A, and the proteins were eluted using Buffer A containing 250 mM of imidazole. The eluted proteins were dialyzed overnight twice, each against 2 L of Buffer A.

### GTPase assay

The release of free inorganic phosphate was quantified using a Malachite Green phosphate assay kit (BioAssay Systems). The enzymatic reaction was conducted in a 100 μL mixture containing 20 mM Tris-HCl (pH 7.5), 400 mM KCl, 5 mM MgCl_2_, 5 mM BME, and 1 mM GTP, along with 1 μM protein, and incubated at 37°C for 2 h. The reaction was halted by adding 20 μL of Working Reagent to 80 μL of the sample solution, followed by a 30-min incubation at room temperature for color development. Optical absorbance was then measured at 620 nm using a Multiskan GO microplate spectrophotometer (Thermo Fischer Scientific).

### Sucrose density gradient sedimentation

Wild-type and *bipA*-deleted strains were inoculated in LB or LB broth supplemented with Kan. The cultures were grown at 37°C or 20°C until the OD_600_ reached 0.5. Subsequently, the cultures were treated with Cm and further incubated at 37°C or 20°C for 3 min. After the Cm treatment and incubation, 50 mL of the cells were harvested at 3,000 rpm for 10 min and resuspended in 0.5 mL of Buffer BP (20 mM Tris-HCl [pH 7.5], 10 mM MgCl_2_, 100 mM NH_4_Cl, and 5 mM BME) for polysome analysis or Buffer BS (20 mM Tris-HCl [pH 7.5], 1 mM MgCl_2_, 100 mM NH_4_Cl, and 5 mM BME) for subunit profiling. The cells were treated with 5 μL of lysozyme and subjected to three freeze-thaw cycles for lysis. The cleared cell lysates were obtained after centrifugation in a Beckman ultracentrifuge tube. For polysome analysis, 320 μg of RNA was loaded onto a 10 mL 5%–40% sucrose gradient in Buffer BP, while for subunit profiling, 160 μg of RNA was loaded onto a 10 mL 5%–25% sucrose gradient in Buffer BS. The cell lysates were fractionated by ultracentrifugation at 4°C for 2.5 h for polysome profiling or 3.5 h for subunit profiling at 37,000 rpm [27].

### Bile salt sensitivity test

Overnight cultures incubated at 37°C were diluted 1:100 in fresh medium and grown at 37°C until they reached an OD_600_ of 0.5. The culture was then diluted to an OD_600_ of 0.2 and subjected to serial dilutions down to 10^−2^. Subsequently, 2.5 μL of the diluted samples were streaked onto bile salt gradient agar plates with concentrations ranging from 2^0^ to 2^7^ g/L.

### Lipopolysaccharide extraction

Colonies grown on LB agar plates incubated at 37°C for 24 h or at 20°C for 2 weeks were scraped into 10 mL of phosphate-buffered saline (PBS, pH 7.2). Based on the OD_600_ reading of the 10 mL suspension, a cell suspension was prepared and harvested at an OD_600_ of 0.5. LPS extraction was performed following the method described by Kenyon *et al.* [38]. The cells were resuspended in 200 μL of TAE buffer (40 mM Tris, 20 mM acetic acid, 1 mM EDTA [pH 8.0]) and 400 μL of LPS lysis buffer (50 mM Tris, 100 mM SDS, 0.128 mM NaCl). Subsequently, 600 μL of phenol solution was added to each sample, and the samples were vortexed for 10 s. The mixtures were incubated at 65°C for 15 min. After cooling to room temperature, the samples were centrifuged at 13,000 rpm for 10 min. LPS was extracted from the aqueous phase and concentrated through ethanol precipitation. The resulting residue was then dissolved in 20 mM Tris-HCl (pH 6.8) and sequentially treated with DNase I, RNase A, and Proteinase K. LPS was re-extracted from the aqueous phase through ethanol precipitation and resuspended in 40 μL of distilled water. LPS samples were analyzed using 15% SDS-PAGE or 16% tricine-SDS-PAGE and visualized with silver staining, as previously described [39].

### Crystal violet staining of the biofilm

The wild-type and *bipA*-deleted strains were grown overnight in LB or LB broth containing Kan at 37°C. The overnight cultures were inoculated 1:100 into a fresh medium in either glass or polypropylene tubes and incubated at 37°C for 24 h. The cultures in glass tubes were incubated at 20°C for 48 h, while those in polypropylene tubes were incubated for 96 h. After incubation, the glass or polypropylene tubes were rinsed with a saline solution and stained with 0.1% crystal violet. The tubes were washed three times with distilled water, and the crystal violet was then dissolved in 95% ethanol. The absorbance was measured at 540 nm.

### Swimming motility assays

The cultures with an OD_600_ of 0.5, incubated at 37°C, were concentrated 10-fold by centrifugation, and 5 μL of the suspension was injected onto an LB plate containing 0.3% Bacto agar for the swimming assay. The incubation times for the wild-type and *bipA*-deleted strains were as follows: *E. coli* for 18 h, SL1344 and 14028 for 4 h, and LT2 for 8 h at 37°C; and *E. coli* for 144 h, SL1344 and 14028 for 22 h, and LT2 for 72 h at 20°C. The migration speed was assessed by measuring the distance traveled from the injection site. The plates were photographed using the Azure C200 Gel Imaging System (Azure Biosystems).

### Transmission electron microscopy

Overnight cultures were inoculated 1:100 into fresh LB medium and cultured statically at 37°C for 1 d or at 20°C for 3 d. The cells were collected by centrifugation at 3,000 rpm and resuspended in PBS. A 10 μL sample was placed on a 200-mesh formvar/carbon-coated copper grid for 10 min and then blotted with filter paper. The grid containing the sample was washed with PBS and stained with 2% phosphotungstic acid for 2 s. The cells were then examined using transmission electron microscopy (TEM, H-7600, HITACHI), and images were captured at 12,000× magnification.

### Human cell lines and culture conditions

Human colorectal intestinal epithelial carcinoma cells (HCT116) were cultured in McCoy’s 5A medium (ATCC), supplemented with 10% (v/v) heat-inactivated fetal bovine serum (Avantor) and 1% (v/v) penicillin-streptomycin (Gibco). The cells were cultured at 37°C in a humidified atmosphere with 5% CO_2_.

### Cell invasion assay

Wild-type strains were transformed using pACYC177, while the *bipA*-deleted strains were transformed with either pACYC177 or pACYC177(Se)BipA. The transformants were cultured at 37°C or 20°C until they reached an OD_600_ of 0.6 and then harvested through centrifugation. The pellets were washed, resuspended in PBS, and mixed with HCT116 cells at a multiplicity of infection (MOI) of 100. After incubation under 5% CO_2_ at 37°C for 1 h, the extracellular bacteria were eliminated by treating the cells with gentamicin (50 μg/mL) for 2 h under the same conditions. The cells were rinsed with PBS, and intracellular bacteria were released using 0.25% sodium deoxycholate. The number of intracellular bacteria was determined by measuring viable counts on LB agar plates.

### *In vitro* cell viability test

The bacterial cells in the cell invasion assay were cultured overnight at 37°C. HCT116 cells were initially plated at a density of 1 × 10^4^ cells per well and incubated for 24 h. Before the experiment, the growth medium (DMEM) supplemented with antibiotics was replaced with antibiotic-free DMEM. The HCT116 cells were then infected with bacterial cells at a MOI of 100 and incubated for 1, 2, 3, and 4 h. After infection, the HCT116 cells were washed with antibiotic-free DMEM containing 50 μg/mL gentamicin and PBS. The cells were then detached using trypsin-EDTA, and the viable and dead cell counts were determined using trypan blue staining.

### Animal studies

Mouse feeding and experimental procedures were conducted as previously described [40]. Specific pathogen-free 6-week-old female BALB/c mice (*n* = 70 for the mouse survival assay, *n* = 30 for the organ invasion assay). The mice were intraperitoneally infected with 10^4^ colony-forming units (CFUs) of *S*. Typhimurium strains in 100 μL of PBS and euthanized after 3 d. The spleen, liver, and mesenteric lymph nodes were aseptically removed, and viable intracellular bacterial cells were quantified as previously described [41]. The log-rank (Mantel-Cox) test was used to analyze the difference in survival rates [42].

### Statistical analysis

Individual data points are represented by white circles, with error bars indicating the standard deviation (SD) from three independent repetitions, otherwise specified. Data were analyzed using an unpaired two-tailed *t*-test. Significance levels were defined as follows: NS (not significant), *p < 0.05, **p < 0.01, ***p < 0.001.

## Results

### The *bipA* deletion in *S*. Typhimurium leads to defective growth at low temperatures

**Fig 1:**
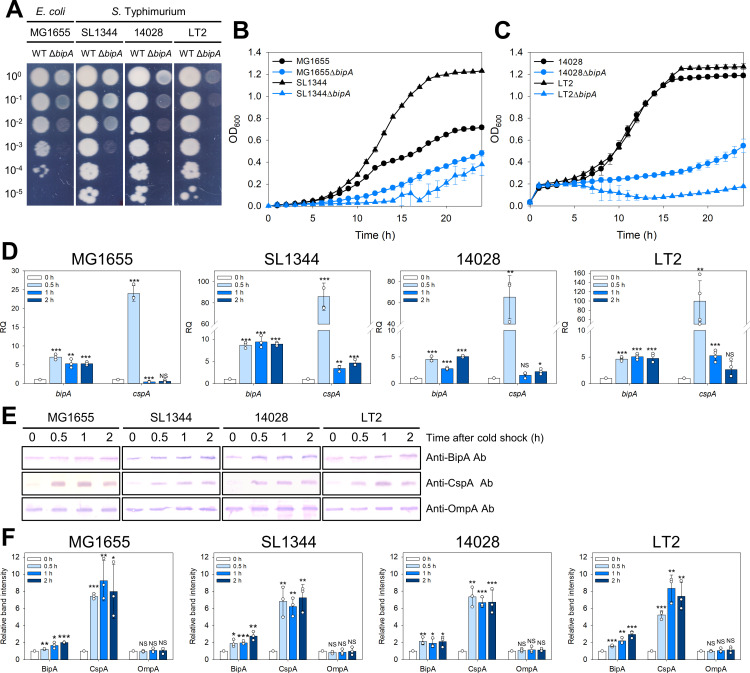
Deletion *of bipA* in *E. coli* and *S.* Typhimurium results in a growth defect at low temperatures. The growth of wild-type and *bipA*-deleted strains of *E. coli* (MG1655) and *S*. Typhimurium (SL1344, 14028, and LT2) was evaluated at 20°C. (A) Overnight cultures were diluted to an OD_600_ of 0.2 in the same medium and further diluted from 10^−1^ to 10^−5^-fold dilutions. Three microliters of the diluted samples were spotted on LB agar plates and incubated at 20°C. (B, C) Cells grown at 37°C to an OD_600_ of 0.5 were inoculated into fresh LB medium and incubated at 20°C for 24 h. Error bars represent the SD. (D) Upregulation of *bipA* transcription after cold shock. The transcript levels of *bipA* and *cspA* were determined using qRT-PCR for the wild-type *E. coli* and *S*. Typhimurium strains at 0, 0.5, 1, and 2 h after cold shock. The relative quantities of the transcripts were calculated against the mean of the reference gene (*rrsA*) and normalized by those obtained for the cells at 0 h. (E) Western blot analyses and (F) densitometry-based quantification of the BipA, CspA, and OmpA protein levels after cold shock were conducted. Samples obtained at the indicated time points were subjected to western blot analysis, and representative images from triplicate experiments are shown. Band intensities were measured using ImageJ software. M, PageRuler-prestained protein ladder (Thermo Fisher Scientific).

Although the *bipA* gene is not essential for optimal growth conditions, previous studies have indicated its critical role in the cold-shock response, enabling cells to adapt to low temperatures in *E. coli* [27]. Thus, the *bipA* gene in the three *S*. Typhimurium strains—SL1344, 14028, and LT2—were deleted to determine whether BipA was required for the normal growth of *Salmonella* strains at low temperatures. As shown in Fig 1A, the *bipA*-deleted *E. coli* (MG1655Δ*bipA*) and *S*. Typhimurium strains exhibited growth defects at low temperatures compared to their wild-type counterparts. Notably, the *bipA*-deleted *S.* Typhimurium LT2 (LT2Δ*bipA*) showed the most significant defects among the *S.* Typhimurium strains at low temperatures (Fig 1A). To quantitatively compare the growth between the strains, growth was monitored by measuring the optical density, consistently showing defective growth of the *bipA*-deleted cells (Fig 1B and 1C). However, when grown at 37°C, both wild-type and *bipA*-deleted strains exhibited similar growth in liquid and solid media, without noticeable differences (S1A-S1C Fig).

To further investigate BipA-dependent growth at low temperatures, we conducted qRT-PCR and western blot analyses to assess the transcriptional and translational changes of BipA following cold shock. As shown in Fig 1D, exposure to cold shock triggered a 4.57- to 8.67-fold increase in *bipA* mRNA levels within 0.5 h in both the *E. coli* and *S*. Typhimurium strains. Additionally, we assessed *cspA* mRNA levels as a positive control, revealing significant increases of 24.02-fold in *E. coli* and 65.30- to 99.85-fold in *S*. Typhimurium strains after cold shock. These increases subsequently returned to the baseline due to auto-repression, consistent with previous findings [43]. The increased expression level of *bipA* transcripts was further confirmed by western blot analysis, which revealed increased BipA expression at the protein level (Fig 1E). Densitometric analysis showed a 2.00- to 2.95-fold increase in BipA protein levels across all strains (Fig 1F). As previously demonstrated [27], the cold-inducibility of *bipA* expression likely involves cAMP-CRP regulation, given the presence of a consensus CRP-binding site upstream of the *SebipA* promoter (S1D Fig). These findings indicate that while BipA expression is moderately induced at low temperatures compared to CspA, its expression is crucial for the growth of both *E. coli* and *S*. Typhimurium under low-temperature conditions and may be regulated by a conserved mechanism in both strains.

### The GTPase activity of BipA is essential for normal growth at low temperatures

**Fig 2:**
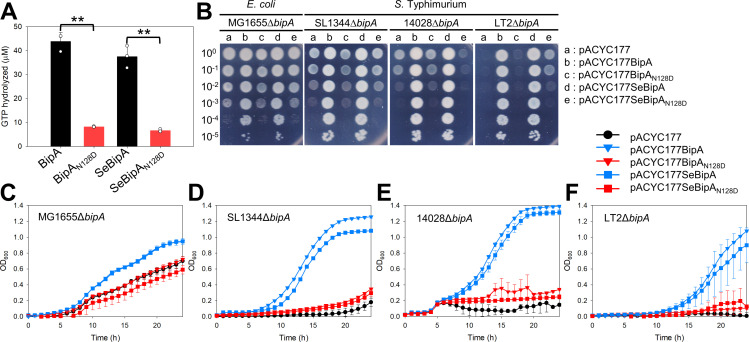
Reduced GTPase activity of BipA_N128D_. (A) GTPase assays were conducted using the wild-type and mutant BipA proteins of *E. coli* and *S*. Typhimurium. The GTP hydrolysis reaction was conducted as described in the Materials and Methods section. (B) Cross-complementation of the *bipA*-deleted strains restored the growth defect. The *bipA-*deleted *E. coli* and *S*. Typhimurium strains were transformed with the plasmid as shown. The transformants were spotted and grown at 20°C, as described in Fig 1. (C–F) The growth of transformants at 20°C was assessed as outlined in Fig 1.

Previous studies on *E. coli* BipA revealed that GTPase activity is essential for normal growth and ribosome association at low temperatures [27]. To investigate the GTPase-dependent function of *S*. Typhimurium BipA (SeBipA), we introduced an inhibitory mutation (Asn128 to Asp) in SeBipA, which disrupts hydrogen bond formation with the 2-amino group of the guanine ring [1]. The *bipA* gene sequences were identical across the three *S*. Typhimurium strains—SL1344, 14028, and LT2. Moreover, primary sequence alignment showed that *E. coli* and *S*. Typhimurium BipA proteins share 96% identity and 99% similarity, with conservation of the Asn128 residue in both proteins (S2A Fig). We constructed the mutant clones pET28SeBipA_N128D_ and pACYC177SeBipA_N128D_ using pET28SeBipA and pACYC177SeBipA as templates. To compare the GTPase activity of the wild-type and mutant BipA, His_6_-tagged BipA proteins from *E. coli* and *S*. Typhimurium were overexpressed and purified, as described in the Materials and Methods section. *In vitro* GTPase assays using these purified proteins revealed that the mutant BipA exhibited approximately 5-fold lower GTPase activities than the wild-type BipA (Fig 2A).

To determine whether the expression of the mutant BipA could restore the cold sensitivity of MG1655Δ*bipA* and *S*. Typhimurium cells with *bipA* deletions were transformed with pACYC177, pACYC177(Se)BipA, or pACYC177(Se)BipA_N128D_. The transformants were cultured in solid or liquid LB media containing ampicillin at 37°C or 20°C. At 20°C, *E. coli* and *S*. Typhimurium cells harboring the mutant plasmids pACYC177BipA_N128D_ or pACYC177SeBipA_N128D_ failed to restore the cold sensitivity, while the cells complemented with the wild-type did (Fig 2B–2F). Western blot analysis showed no significant difference in the expression levels of the wild-type and mutant BipA proteins across the four mutant strains (S2B Fig). This indicates that the inhibitory effect of SeBipA_N128D_ in 14028Δ*bipA* and its promotive effect in LT2Δ*bipA* strains were not attributable to differences in expression levels. At 37°C, all transformants exhibited normal growth without defects (S2C Fig). These findings indicate that the GTPase activity of BipA is essential for its function and that *E. coli* BipA can replace SeBipA, and *vice versa*. Furthermore, the growth-defective phenotype resulting from *bipA* deletion was not due to a polar effect.

### The critical role of BipA in the ribosome assembly of *S*. Typhimurium

**Fig 3:**
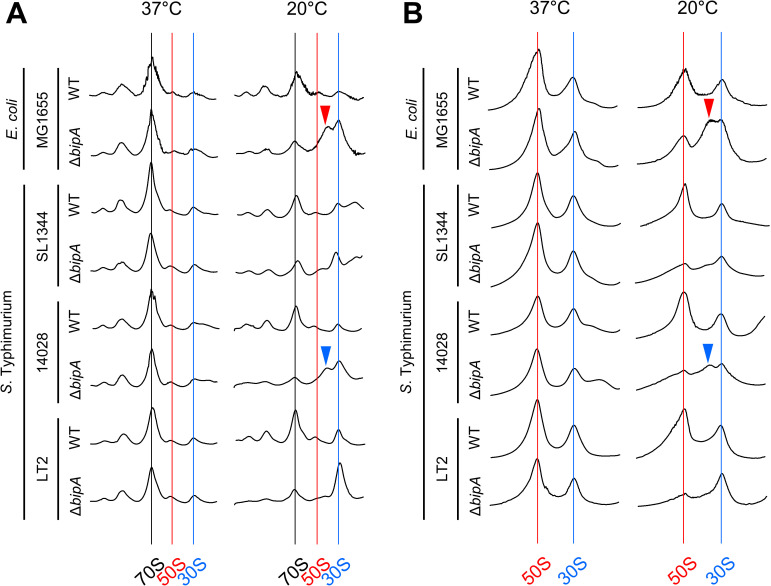
Ribosome assembly defects in the *bipA*-deleted strains at low temperatures. (A) Polysome profiles and (B) subunit profiles of wild-type and *bipA*-deleted strains of *E. coli* and *S*. Typhimurium. Wild-type and mutant strains were inoculated into LB or LB broth containing Kan (50 μg/mL). Cells were grown at 37°C or 20°C until the OD_600_ reached 0.5 and then harvested by centrifugation at 3,000 rpm for 10 min. Cleared cell lysates were prepared, and sucrose gradient sedimentation was performed as described in the Materials and Methods section. The red and blue arrowheads indicate abnormal 50S ribosome particles in the *E. coli* and *S*. Typhimurium strains.

In *E. coli*, *bipA*-deleted cells accumulate abnormal 50S ribosomal subunits at low temperatures. Wild-type BipA associates with both 70S ribosomes and 50S ribosomal subunits in a GTP-dependent manner, whereas BipA_N128D_ does not exhibit this association [27]. To investigate the interaction between SeBipA and the 50S ribosome and its implications, we conducted polysome and subunit profiling analyses using sucrose gradient sedimentation. Cleared cell lysates were prepared and subjected to ultracentrifugation, as described in the Materials and Methods section. Polysome analyses of cells grown at 37°C revealed that all strains had normal polysomes and ribosomal subunits, with or without BipA. However, in MG1655Δ*bipA* cells cultured at 20°C, the peak of the 50S subunit collapsed, while the peak of the 30S increased (Fig 3A). Consistent with our previous report, abnormal particles (~44S, indicated by the red arrowhead in Fig 3A) accumulated between the 50S and 30S peaks [27]. Among *S*. Typhimurium strains SL1344, 14028, and LT2, *S*. Typhimurium 14028Δ*bipA* exhibited a pattern most similar to MG1655Δ*bipA* (indicated by the blue arrowhead in Fig 3A). Conversely, the SL1344Δ*bipA* and LT2Δ*bipA* strains did not show an abnormal 50S ribosomal peak; instead, there was a significant increase in the 30S peak compared to the wild-type. Ribosomal subunits analyzed under lower sucrose gradient concentrations revealed highly superimposable subunit profiles between the wild-type and mutant strains at 37°C (Fig 3B). However, similar to the polysome profiling results, a consistent abnormality was observed in the 50S subunits, either through the accumulation of ~44S particles or a diminished quantity. Notably, *bipA*-deleted *S*. Typhimurium strains exhibited a reduced abundance of intact 50S subunits at 20°C compared to the MG1655Δ*bipA* strain. Unlike *E. coli* MG1655, these *S*. Typhimurium strains possess rRNA genes that contain one or two intervening sequences in the 23S rRNA portion (S3 Fig). These intervening sequences are excised by RNase III during maturation, resulting in ribosomes with two or three cleaved segments of 23S rRNA [44]. While cleavage within the 23S rRNA does not affect ribosomal function, it accelerates ribosomal degradation by creating more targets for ribonucleases [45]. Consequently, in the absence of BipA, nascent 50S ribosomes likely undergo structural instability, making them more susceptible to degradation. This ultimately results in significantly low levels of intact 50S ribosomal subunits in *bipA*-deleted *S*. Typhimurium strains at low temperatures (Fig 3B).

**Fig 4:**
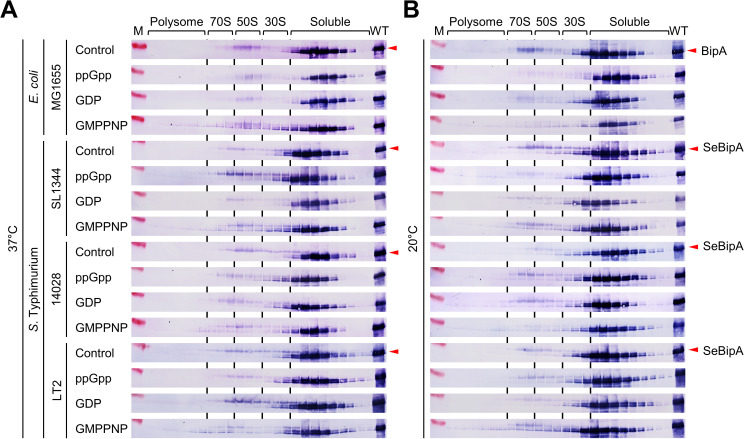
Nucleotide-dependent ribosome association of BipA. Wild-type *E. coli* (MG1655) and *S*. Typhimurium (SL1344, 14028, and LT2) strains were cultured at (A) 37°C or (B) 20°C, as outlined in Fig 3. Cell pellets were resuspended in buffer BP containing ppGpp, GDP, or GMPPNP, each at a final concentration of 100 μM. Sucrose gradient sedimentation was conducted as described in the Materials and Methods section. Fractions from polysome to free RNAs in polysome profiling were subjected to western blot analysis using anti-BipA antibodies. The control “WT” indicates wild-type MG1655 cells harboring pACYC177(Se)BipA. M, molecular marker.

Previous studies have shown that His_6_-tagged SeBipA binds to the 70S ribosome in the presence of GTP. Furthermore, under stringent response conditions or the presence of ppGpp, His_6_-SeBipA exhibited an increased affinity for the 30S ribosomal subunit [46]. Therefore, we further investigated how SeBipA modulates its ribosome binding mode, depending on the nucleotides present. To precisely determine the ribosome binding mode of SeBipA, we mixed wild-type cells cultured at 37°C or 20°C with ppGpp (a stringent response alarmone), GDP, and GMPPNP (a nonhydrolyzable analog of GTP) at a final concentration of 100 μM. Subsequently, we performed sucrose gradient sedimentation and western blotting to locate the endogenously expressed SeBipA proteins in the polysome profiling fractions. Both BipA and SeBipA bound to 70S ribosomes and 50S ribosomal subunits at both temperatures in the presence of GMPPNP, indicating a temperature-independent association (Fig 4A and 4B). For the 30S ribosome association, both BipA and SeBipA were detected in the trail fraction near the soluble portion, indicating that this association may not be GMPPNP-specific and requires further investigation. Notably, adding GDP or ppGpp did not enhance the interaction between *E. coli* BipA and ribosomal fractions at either temperature. Conversely, SeBipA from pathogenic *S*. Typhimurium SL1344 and 14028 appeared to cosediment with polysomes and 70S ribosomes in the presence of ppGpp and GDP at low temperatures. This suggests that SeBipA may have a different binding mechanism than *E. coli* BipA. Nevertheless, our findings indicate that the specific interaction of BipA proteins with 70S and 50S ribosomes is GMPPNP-dependent. Although BipA binds to 50S ribosomal subunits at 37°C, ribosome biogenesis occurs without defects, indicating that BipA is dispensable.

### Increased bile salt sensitivity and LPS synthesis defects in *bipA*-deleted cells at low temperatures

Exposure to BPI with LPS decomposition activity results in an approximately 7-fold upregulation of *bipA* expression in *S*. Typhimurium [5], and its deletion makes bacterial cells more sensitive to BPI [26]. This suggests that BipA may influence the LPS structure or composition in response to BPI. Our previous findings further support this, showing that MG1655Δ*bipA* cells accumulate an abnormal lipid A-core oligosaccharide (lipooligosaccharides, LOS) at low temperatures [28]. This LPS perturbation, whether due to *bipA* deletion or other outer membrane damage, is known to signal through the Rcs pathway [47]. The outer membrane protein RcsF initiates this process by detecting stress signals, which are then transmitted through the inner membrane proteins RcsC-RcsD to the response regulator RcsB through a phosphorelay system [48]. This signaling cascade ultimately regulates the expression of various genes. The phosphorylated RcsB can form a heterodimer with RcsA or a homodimer with itself. The former activates the transcription of the *cps* gene cluster for colanic acid synthesis and represses the expression of *flhDC*, which encodes the primary regulator of flagella biosynthesis genes [49,50]. Conversely, the latter dimer stimulates the expression of *rprA*, which encodes a negative regulatory sRNA of curli and cellulose production [51]. Therefore, the Rcs two-component regulatory system is a key signaling pathway for protecting cells from environmental challenges and intrinsic sources of envelope stress.

**Fig 5:**
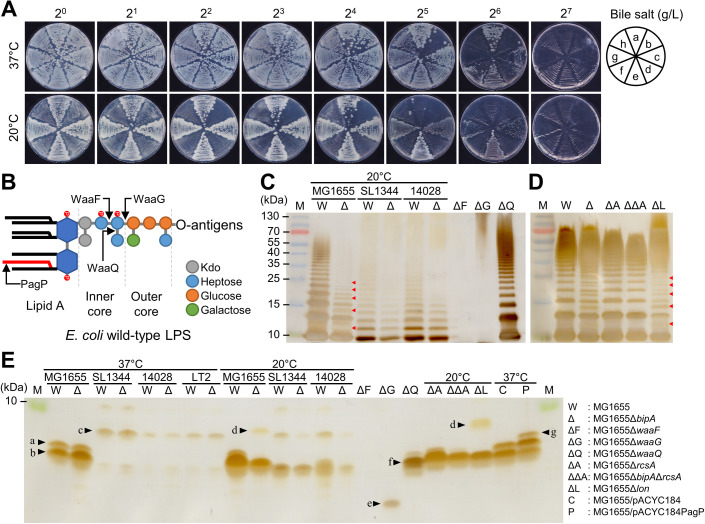
Analysis of bile salt sensitivity and LPS profiles in the wild-type and *bipA*-deleted strains of *E. coli* and *S*. Typhimurium. (A) Bile salt-sensitive phenotypes of (a) MG1655, (b) MG1655Δ*bipA*, (c) SL1344, (d) SL1344Δ*bipA*, (e) 14028, (f) 14028Δ*bipA*, (g) LT2, and (h) LT2Δ*bipA* at 37°C or 20°C. Overnight cultures were diluted to an OD_600_ of 0.2 in the same medium and further diluted 10^−2^-fold. Two and a half microliters of the diluted samples were spread onto LB agar plates containing a gradient concentration of bile salt and incubated overnight at 37°C or 20°C for 1 week. (B) Schematic LPS structure of *E. coli* K-12. The action sites of WaaF, WaaG, WaaQ, and PagP were indicated. Kdo, 3-deoxy-d-manno-oct-2-ulosonic acid. (C) LPS profile analysis of the *E. coli* and *S*. Typhimurium strains. LPS was extracted and analyzed using SDS-PAGE followed by silver staining, as described in the Materials and Methods section. M, molecular marker. (D) LPS profile analysis of colanic acid-overproducing strains. LPS samples were extracted from cells grown on LB agar plates as in (C). (E) Lipid A-core oligosaccharide profile analysis. Twenty-five-fold diluted LPS samples were separated by tricine-SDS-PAGE followed by silver staining.

Therefore, we examined how *bipA* deletion in each strain affects cell surface structures, such as LPS, capsule, biofilm, and flagella, at both temperatures. To determine whether BipA of *S*. Typhimurium strains is involved in LPS biosynthesis, we streaked wild-type MG1655 and the three *S*. Typhimurium strains (SL1344, 14028, and LT2), along with their *bipA*-deleted strains on solid media containing bile salt at concentrations ranging from 2^0^ to 2^7^ g/L. The plates were incubated at either 37°C or 20°C. Bacterial strains with LPS defects are known to be more susceptible to bile salts [52,53]; thus, we indirectly assessed the LPS status by examining bile salt sensitivity. As shown in Fig 5A, while BipA is dispensable for ribosome synthesis at 37°C, the colony formation of MG1655Δ*bipA* cells was inhibited at a bile salt concentration of 2^5^ g/L at 37°C. Conversely, wild-type cells failed to grow at a bile salt concentration of 2^7^ g/L. Note that the concentration of bile salts in human bile varies depending on the source and can reach to 3.5% in the duodenum and ≤0.005% in the gastrointestinal tract [54]. Among the *S.* Typhimurium strains, LT2 was more sensitive to bile salts than the other two strains, and the *bipA*-deleted cells were more sensitive than the wild-type cells. At 20°C, the four *bipA*-deleted strains lost nearly all growth ability, failing to form colonies at a bile salt concentration of 2^2^ g/L, while the wild-type cells continued to grow at bile salt concentrations ranging from 2^4^ to 2^5^ g/L. These results indicate that the deletion of *bipA* in both *E. coli* and *S.* Typhimurium significantly influences LPS biosynthesis at 20°C, and, to a minimal extent, at 37°C.

To further investigate LPS abnormalities in *bipA*-deleted cells, we extracted LPS from the wild-type and *bipA*-deleted strains grown on LB agar plates at 37°C or 20°C. The LT2Δ*bipA* cells did not adequately grow at 20°C, resulting in insufficient biomass for LPS extraction. As controls, we extracted LPS from three *E. coli* mutants with deletions in the *waaF*, *waaG*, or *waaQ* genes, which are responsible for adding the second heptose, the first glucose, and the side-branch third heptose, respectively (Fig 5B) [55–57]. The LPS samples were then analyzed using SDS-PAGE or tricine-SDS-PAGE followed by silver staining. In the *waaF-* and *waaG*-deleted strains, which exhibit severe defects in the LPS core oligosaccharide, mature LPS was not observed, whereas the *waaQ* mutant showed a normal LPS ladder (Fig 5C). As shown in S4A Fig, no discernible differences were observed between the wild-type and *bipA*-deleted strains at 37°C. However, at 20°C, a significant reduction in LPS levels was observed in the *bipA*-deleted *E. coli* and *S.* Typhimurium strains. Notably, additional LPS ladder bands were observed between the repeated O-antigen units in the MG1655Δ*bipA* strain (indicated by the red arrowheads in Fig 5C). These bands were absent in the MG1655 strain, suggesting potential alterations in the LOS moiety or variations in the size of the O-antigen unit. It has been reported that when colanic acid, also referred to as the M-antigen (mucoid antigen), is overproduced, the M-antigen can replace the O-antigen on the LPS, resulting in the formation of M-LPS [58,59]. To investigate this possibility, LPS was extracted from the MG1655Δ*rcsA*, MG1655Δ*bipA*Δ*rcsA*, and MG1655Δ*lon* strains and compared with that from MG1655Δ*bipA*. Lon is a protease that degrades RcsA, the activator of the *cps* cluster, and in its absence, colanic acid was overproduced at 20°C (S4B Fig) [49,60]. As shown in Fig 5D, the aberrant LPS ladder was observed in both MG1655Δ*bipA* and MG1655Δ*lon* strains, while no such ladder was observed in the *rcsA*-deleted cells, suggesting the formation of M-LPS. In the case of *S*. Typhimurium strains, the LPS ladders were more closely spaced due to the smaller size of the O-antigen units compared to those of *E. coli* [61].

To further investigate potential perturbations in the LOS moiety, we analyzed the LPS extracts using tricine-SDS-PAGE. An examination of the LOS from the three control mutants showed that the absence of a branched heptose in the inner core did not result in a substantial size difference compared to the LOS from the wild-type cells (band “f” in Fig 5E). However, the absence of five sugars due to the *waaG* deletion significantly reduced LOS size (band “e” in Fig 5E), and the LOS in the *waaF* mutant was barely detectable (Fig 5E).

At 37°C, both MG1655 and MG1655Δ*bipA* cells exhibited two major LOS forms (bands “a” and “b” in Fig 5E), and they are likely to be a hexa-acylated form based on the position of the hepta-acylated form by PagP (band “g”) [62]. Likewise, no significant LOS alterations were observed between the wild-type and mutant *S*. Typhimurium strains. Notably, LOS + 1 O-antigen unit was predominant in *S*. Typhimurium compared to *E. coli* (band “c” in Fig 5E). However, at 20°C, LOS forms without O-antigen unit became prevailing, and their overall amount was diminished in the mutant *S*. Typhimurium.

More interestingly, the larger LOS (band “a”) disappeared in MG1655Δ*bipA* cells grown at 20°C, along with a concomitant reduction in the smaller LOS (band “b”). This overall decrease in LOS levels likely explains the observed reduction in total LPS. Unexpectedly, a band slightly larger than the LOS + 1 O-antigen unit of *S*. Typhimurium appeared (band “d”), which was also observed in MG1655Δ*lon* cells. Considering that the molecular weight of M-antigen (1,106.0 Da) is larger than that of O-antigen in *S*. Typhimurium (672.6 Da), the band “d” likely represents LOS + 1 M-antigen unit [63,64]. Furthermore, it is also possible that the missing band “a” in MG1655Δ*bipA* cells at 20°C was used to synthesize the M-LPS, consistent with the result in Fig 5D.

Taken together, at 20°C, the *bipA-*deleted *S.* Typhimurium produced less LOS, whereas *bipA* deletion in *E. coli* resulted in significant changes in LOS and LPS structures, consequently impairing outer membrane integrity.

### The effect of *bipA* deletion on the capsule and biofilm

To assess changes in capsule synthesis, diluted cell cultures were either spread on LB agar plates or spotted onto MacConkey agar plates. Following incubation at 37°C or 20°C, we examined the morphologies of the colonies and macrocolonies. On LB agar plates, MG1655Δ*bipA* colonies merged with neighboring colonies, resulting in a mucoid appearance at 20°C—a phenomenon not observed in any other strains at either temperature (S5A Fig). Similarly, only MG1655Δ*bipA* cells exhibited a transparent halo around the macrocolony on MacConkey agar plates at 20°C (S5B Fig)*.* Although the regulatory mechanism of the *cps* cluster through the Rcs pathway is conserved in *S*. Typhimurium [65], the *bipA*-deleted *S*. Typhimurium strains, unlike MG1655Δ*bipA* cells, did not exhibit capsule overproduction at either temperature under the specified growth conditions.

**Fig 6:**
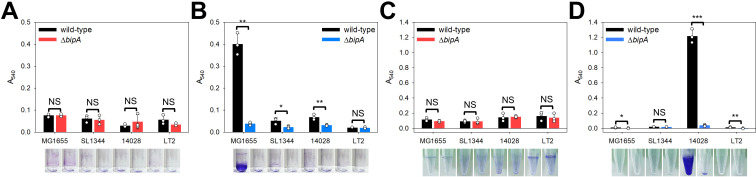
Effect of *bipA* deletion on biofilm formation in *E. coli* and *S*. Typhimurium. Biofilm formation of wild-type *E. coli* (MG1655) and *S*. Typhimurium (SL1344, 14028, and LT2), along with their *bipA*-deleted strains at 37°C or 20°C. Cell culturing and crystal violet staining were performed as described in the Materials and Methods section. The cultures in glass tubes were incubated at (A) 37°C or (B) 20°C. The cultures in Eppendorf tubes were incubated at (C) 37°C or (D) 20°C.

The Rcs signaling pathway enhances the production of the small regulatory RNA RprA [66]. RprA represses the translation of *csgD*, which encodes a positive regulator of curli synthesis and cellulose production, resulting in decreased biofilm formation [67,68]. Additionally, it has been reported that the overproduction of colanic acid inhibits biofilm formation by reducing surface adhesion [69]. Therefore, we compared the biofilm formation abilities of the wild-type and *bipA*-deleted strains. Biofilm formation on a glass tube, representing a hydrophilic surface, was assessed using the crystal violet staining method as described in the Materials and Methods section. As shown in Fig 6A, none of the wild-type or mutant strains formed substantial biofilm at 37°C, whereas biofilm formation in the wild-type *E. coli* strain increased approximately 5.31-fold at 20°C compared to 37°C. Notably, MG1655Δ*bipA* cells showed a significant reduction in biofilm formation (0.09-fold) (Fig 6B). The SL1344Δ*bipA* and 14028Δ*bipA* strains exhibited a 0.44-fold and 0.46-fold decrease in biofilm-forming ability, respectively, while no significant difference was observed in the LT2 strain (Fig 6B).

Given that *S*. Typhimurium biofilms preferentially attach to hydrophobic surfaces [70], we conducted a parallel experiment using polystyrene tubes with hydrophobic surfaces. Consistent with the results observed with glass tubes, minimal biofilm formation was observed at 37°C in both the wild-type and *bipA*-deleted strains, with no significant difference in the biofilm-forming ability between them (Fig 6C). Note that conventional laboratory conditions for *Salmonella* biofilm formation involve low osmolarity (low- salt LB) and a temperature of 28°C, which differ from our experimental conditions. These conditions promote RpoS-dependent activation of biofilm-related genes such as *csgD*, *adrA*, and *agfA* [71,72]. However, at 20°C, the wild-type 14028 cells formed a substantial amount of biofilm, which significantly reduced in the absence of BipA (0.03-fold) (Fig 6D).

These findings indicate that the distinct cell surface properties of *E. coli* and *S*. Typhimurium result in opposite patterns of bacterial attachment depending on whether the surface is hydrophobic or hydrophilic. Nevertheless, *bipA* deletion resulted in reduced biofilm formation in both *E. coli* and *S*. Typhimurium 14028.

### Reduced motility of *bipA*-deleted strains with fewer flagella

Given that the Rcs pathway inhibits the expression of *flhDC*, the master regulator of flagella biosynthesis, we investigated how *bipA* deletion affects motility and flagella synthesis in *S*. Typhimurium strains.

**Fig 7:**
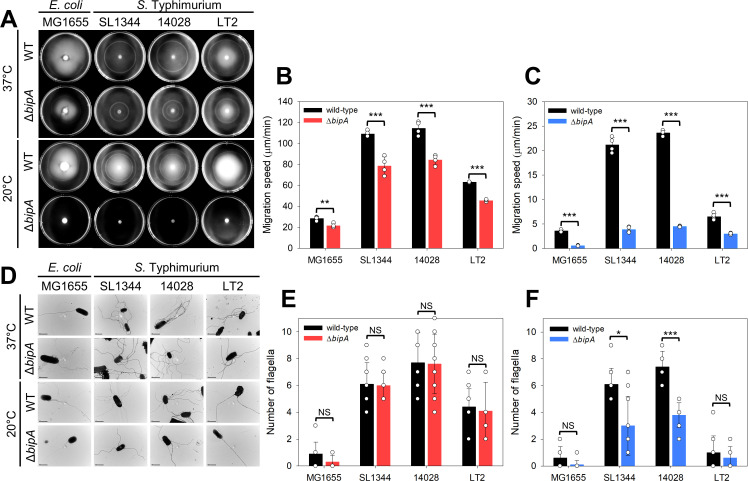
Flagella-mediated motility affected by *bipA* deletion in *S*. Typhimurium. (A) Swimming motility assays of wild-type *E. coli* (MG1655) and *S*. Typhimurium (SL1344, 14028, and LT2), along with their *bipA*-deleted strains. Overnight cultures were diluted 10^−2^-fold in fresh medium and incubated until the OD_600_ reached 0.5. The cultures were then concentrated 10-fold and injected onto LB agar plates, followed by incubation at 37°C or 20°C. (**B** and C) The migration speeds of the wild-type and *bipA*-deleted strains were measured at (B) 37°C or (C) 20°C by recording the distance traveled per incubation time. Error bars represent the SD from four independent replicates and each data point is indicated by a white circle. (D) Fewer flagella were observed in *bipA*-deleted pathogenic *S*. Typhimurium strains at low temperatures. Overnight cultures were diluted 10^−2^-fold in fresh medium and incubated at 37°C for 1 d or 20°C for 3 d. The cells were harvested at 3,000 rpm to minimize flagella loss, deposited onto carbon-copper grids, and negatively stained. TEM was used to visualize the flagella of the wild-type and *bipA*-deletion strains. Flagella count was quantified from 10 individual cells grown at (E) 37°C or (F) 20°C, represented in the bar graphs.

To assess swimming motility, cells in the early exponential phase were inoculated onto 0.3% agar plates and incubated at 37°C or 20°C. As shown in Fig 7A and 7B, the wild-type pathogenic *S*. The Typhimurium SL1344 and 14028 strains exhibited significantly faster motility at 37°C than the *E. coli* MG1655 and *S*. Typhimurium LT2 strains. In all four strains, *bipA*-deleted strains resulted in a moderate reduction in motility (0.72–0.76-fold) (Fig 7B). While the overall swimming motility decreased at 20°C compared to 37°C, the two pathogenic strains maintained higher migration speeds than their nonpathogenic counterparts (Fig 7A and 7C). Notably, the reduction in the migration speed of the *bipA*-deleted strains, particularly, SL1344Δ*bipA* and 14028Δ*bipA*, became more pronounced at 20°C (0.16–0.46 fold) compared to 37°C (Fig 7C).

This flagella-mediated motility was further confirmed through a swarming assay. As shown in S6A Fig, neither the wild-type nor the mutant *E. coli* strains exhibited motility at either temperature. Consistent with the swimming motility results, the two pathogenic *S*. Typhimurium strains exhibited higher motility than the attenuated LT2 strain. However, the mutant *S*. Typhimurium strains completely lost motility at 20°C, highlighting the role of BipA in flagella function.

To correlate the reduced mobility with the diminished expression of flagella, the flagellated states of the respective strains were examined using TEM. Cells cultured in the stationary phase at 37°C or 20°C were stained with phosphotungstic acid on a carbon-copper grid and analyzed using TEM, as described in the Materials and Methods section. As shown in Fig 7D and 7E, the SL1344 and 14028 strains exhibited approximately 6–8 flagella on their surfaces at 37°C, while LT2 had fewer (approximately 4), and the MG1655 had the least (only 1 flagellum). At 20°C, the flagella counts for the four strains followed the same pattern; however, the mutant strains produced half the number of flagella compared to the wild-type strains (Fig 7F). At 37°C, there was no significant difference in flagella count between the wild-type and mutant strains (Fig 7D and 7E). This finding was further confirmed by western blot analysis targeting the deflagellated FliC proteins, as outlined in S1 Supporting Information. In the MG1655 and LT2 strains, the FliC protein was undetectable in the deflagellated soluble fraction at both temperatures, likely due to the lower flagella count. Conversely, a similar amount of deflagellated FliC protein was detected in both wild-type and mutant SL1344 and 14028 strains at 37°C. However, at 20°C, these FliC proteins diminished in the SL1344Δ*bipA* and 14028Δ*bipA* strains (S6B Fig).

These phenotypes were further validated by analyzing the expression levels of several key flagella-related genes (*flgM*, *fliE*, *fliK*, *fliM*, *fliS*, and *fliZ*) in all four strains at both temperatures using qRT-PCR. The deletion of *bipA* significantly reduced gene expression in all strains, particularly at 20°C (S7 Fig). Given the phenotypic effects of *bipA* deletion in *S*. Typhimurium, it is likely that the outer membrane damage from reduced LPS in *bipA*-deleted strains results in reduced flagella production, thereby diminishing motility.

### Effect of *bipA* deletion on the pathogenicity of *S*. Typhimurium SL1344 and 14028 strains

**Fig 8:**
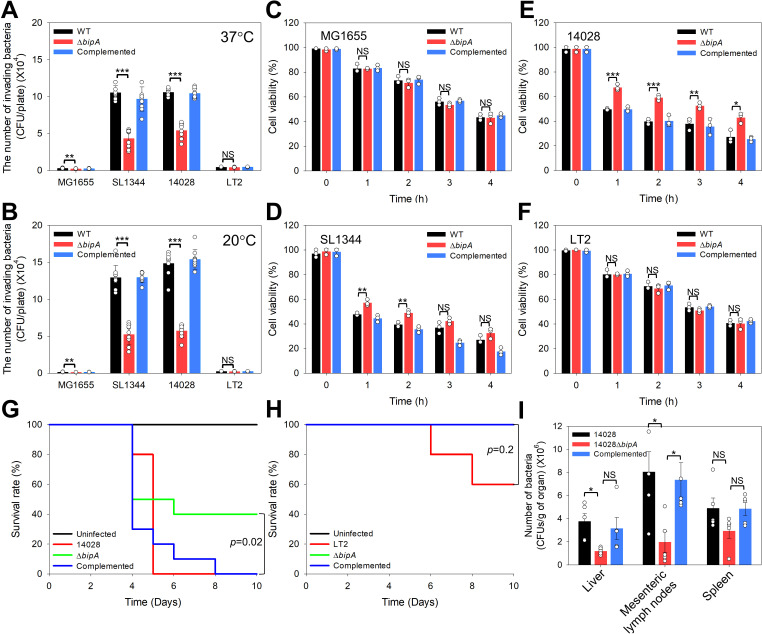
Deletion of *bipA* in pathogenic *S*. Typhimurium strains resulted in reduced invasion into HCT116 cells. Effect of *bipA* expression on epithelial cell invasion by *E. coli* (MG1655) and *S*. Typhimurium strains (SL1344, 14028, and LT2). Epithelial cells (HCT116) were infected with the wild-type and *bipA*-deleted strains of *E. coli* and *S*. Typhimurium at an MOI of 100, and the number of intracellular bacteria was counted as CFUs at (A) 37°C or (B) 20°C. Error bars represent the SD from nine independent replicates and each data point is indicated by a white circle. (C–F) Effect of *bipA* expression on *E. coli* (MG1655) and *S*. Typhimurium strains (SL1344, 14028, and LT2) epithelial cell viability. HCT116 cells were infected with *E. coli* and *S*. Typhimurium cultures from (B) at an MOI of 100. Cell viability was determined by counting viable cells using the trypan blue staining assay at the indicated time points. (G) and (H) Survival rates and durations of BALB/c mice infected with *S*. Typhimurium (14028 and LT2), *bipA*-deleted strains, and PBS as a control (uninfected) were monitored for 10 d (*n* = 10 per group). (I) Colonization assay in BALB/c mice (*n* = 5 per group) infected with *S*. Typhimurium 14028 and *bipA*-deleted strains, with bacterial CFUs counted in the liver, mesenteric lymph nodes, and spleen. Data were expressed as the mean ± standard error of the mean from at five independent experiments, with individual data points indicated by white circles.

In many pathogens, flagella-related phenotypes are closely linked to infection processes, such as host cell invasion and virulence in mouse models [40,73–75]. Our findings revealed that *bipA* deletion affected LPS, biofilm, and flagella formation (Figs 5, 6, and 7). To determine whether *bipA* deletion affects the pathogenicity of *S*. Typhimurium, we examined its influence on host cell invasion. HCT116 cells were infected with wild-type (WT; + pACYC177), *bipA*-deleted (Δ*bipA*; + pACYC177), and *bipA*-complemented [Complemented; + pACYC177(Se)BipA] strains cultured at 37°C or 20°C. After infection, gentamicin was used to eliminate bacterial cells that did not infect the host cells. Bacterial invasion was then assessed by measuring the CFUs. As anticipated, neither the *E. coli* nor *S*. Typhimurium LT2 strains showed any invasion of HCT116 cells at either temperature. Conversely, the *bipA*-deleted pathogenic *S*. Typhimurium SL1344 and 14028 strains produced approximately 45% fewer CFUs in HCT116 cells than the wild-type and complemented strains at both temperatures (Fig 8A and 8B). The invasion assay indicated reduced pathogenicity, a finding further supported by an *in vitro* cytotoxicity assay, in which dead and viable cells were differentiated using trypan blue staining. Consistent with the invasion assay results, *E. coli* and *S.* Typhimurium LT2 strains did not significantly affect host cell viability (Fig 8C and 8F). However, the number of viable HCT116 cells increased when infected with the *S*. Typhimurium SL1344Δ*bipA* and 14028Δ*bipA* strains compared to those infected with wild-type or complemented cells (Fig 8D and 8E).

Next, we investigated the role of BipA-related *S*. Typhimurium pathogenicity using a mouse model infected with *S*. Typhimurium 14028 and LT2 strains, which exhibited distinct differences in host cell invasion. Mice infected with either the wild-type or complemented *S*. Typhimurium 14028 strains succumbed to infection within 5 and 8 d, respectively. Conversely, 40% of the mice infected with the 14028Δ*bipA* strain survived 8 d postinfection (Fig 8G). Although the wild-type LT2 strain exhibited a slight mortality rate, *bipA* deletion did not influence the pathogenicity of the *S*. Typhimurium LT2 strain (Fig 8H). We also assessed the bacterial load in the liver, mesenteric lymph nodes, and spleen of the infected mice. The bacterial load in the 14028Δ*bipA* strain was significantly lower than that of the wild-type *S*. Typhimurium 14028 strain across all three organs. Notably, this colonization defect was restored to the wild-type levels upon complementation with *bipA* (Complemented; Fig 8I). These findings clearly demonstrate the role of BipA in regulating the pathogenicity of the *Salmonella* Typhimurium SL1344 and 14028 strains.

## Discussion

In this study, we comprehensively assessed the roles of BipA in ribosome assembly and virulence factors across two different bacterial species, *E. coli* and *S*. Typhimurium, evaluating its impact on pathogenicity of the strain *S*. Typhimurium 14028 strain. In our previous study, we demonstrated that BipA exhibits protein-chaperone activity by refolding denatured proteins. We also reported that the incorporation of the ribosomal protein L6 into the 50S ribosomal subunit in *E. coli* was hindered at low temperatures in the absence of BipA, indicating the role of BipA as a 50S ribosome assembly factor [27]. Our results revealed that the GTP-dependent association of BipA with the 50S ribosome is crucial for ribosome assembly in *S*. Typhimurium at low temperatures. Deletion of *bipA* reduced the levels of the 50S ribosomal subunit and resulted in the accumulation of abnormal 50S ribosomal particles at 20°C (Fig 3). This finding indicates that the role of BipA in ribosome assembly is conserved across various bacterial species.

Notably, while BipA is not essential for ribosome assembly at 37°C, *bipA*-deleted *E. coli* and *S*. Typhimurium cells exhibited increased sensitivity to higher concentrations of bile salts at 37°C. This sensitivity was also evident at lower concentrations of bile salts at low temperatures (Fig 5). Additionally, the *bipA* mutants were more susceptible to the cationic antimicrobial peptide polymyxin B, which targets the outer membrane, at low temperatures compared to wild-type cells (S8 Fig).

As BipA co-localizes with the 70S ribosome in the presence of GMPPNP, it may play a role in the temperature-dependent formation of specialized ribosomes that preferentially translate mRNAs involved in LPS synthesis or modification. Such specialized ribosomes can form through various mechanisms, including rRNA sequence variations, rRNA modification, r-protein modifications, r-protein paralogs, and ribosome-associated proteins [76]. The association of BipA with the ribosome may induce conformational changes in rRNAs and r-proteins, resulting in the formation of ribosomes with altered structural and functional properties. Alternatively, ribosome-associated BipA may directly recruit specific mRNAs to the ribosome, similar to the pro-apoptotic protein Reaper in *Drosophila melanogaster* [77] and the RACK1 protein in *Saccharomyces cerevisiae* [78–80], thereby promoting the specific translation of LPS-related mRNAs at low temperatures.

LPS, a glycolipid present on the cell surface of Gram-negative bacteria, consists of a conserved lipid A anchor, a core oligosaccharide, and an O-antigen. The synthesis of LPS begins at the cytoplasmic side of the inner membrane through the Raetz pathway, which involves a series of conserved enzymatic steps. This pathway initiates with UDP-*N*-acetylglucosamine, which is converted into Lipid IV_A_, then to Kdo_2_-lipid A, eventually forming LOS that includes core sugars. The LOS is flipped to the periplasmic side by the MsbA_2_ flippase. Subsequently, the O-antigen is added by the O-antigen ligase. Finally, the Lpt complex transports the complete LPS molecule to the outer leaflet of the outer membrane [81]. During or after the Raetz pathway, LPS can be further modified by various enzymes that typically alter the phosphate groups, the sugar backbone of lipid A, the core oligosaccharide, and the acyl chains.

In the MG1655Δ*bipA* strain at 20°C, an M-LPS was observed, along with the absence of the larger LOS band (Fig 5C and 5E). Notably, in the *waaG*-deleted strain, only one LOS band was detected, suggesting that the missing band represents a LOS variant with a modified outer core. MG1655 is reported to predominantly contain glycoform I core oligosaccharide [82,83], which likely corresponds to the smaller LOS band (band “b” in Fig 5E). Given that hepta-acylated LOS is 256.42 Da larger than hexa-acylated LOS, and the missing band is positioned between these two forms, it is predicted that this missing LOS species is approximately 120 Da larger than glycoform I. Based on its size and modification position, it is likely glycoform II, which contains an additional *N*-acetylglucosamine attached to the fourth heptose, compared to glycoform I [84]. However, the enzyme responsible for this modification has not yet been definitively identified. Therefore, it is essential to explore the functional relationship between LOS-synthesizing enzymes and BipA at low temperatures.

The Rcs phosphorelay system is activated in response to cell envelope stresses, including defects in LPS, leading to increased transcription of the *cps* gene cluster and *rprA* and repression of the *flhDC* operon [48,50,66,85]. The phenotypic traits observed in the *bipA*-deleted *E. coli*—such as defects in LPS biosynthesis, capsule overproduction, impaired biofilm formation, and reduced flagella-mediated motility—indicate the activation of the Rcs pathway. Typically, perturbations in the cell envelope are detected by the outer membrane lipoprotein RcsF. The activated RcsF then interacts with the inner membrane protein IgaA, disrupting the inhibitory action of IgaA on RcsD. Subsequently, the phosphorelay occurs from RcsC to RcsD to RcsB [85]. This signal transmission can be described as an RcsF-dependent pathway. In S4B Fig, the MG1655Δ*bipA*Δ*rcsF* appear to overproduce capsular polysaccharides, whereas the MG1655Δ*bipA*Δ*rcsA* cells do not. This indicates that the activation of the Rcs pathway in the MG1655Δ*bipA* cells may be mediated in an RcsF-independent manner, while capsule overproduction relies on the RcsA. One potential trigger for activating the Rcs pathway in an RcsF-independent manner is the overexpression of *djlA*, which encodes the cochaperone DnaJ homolog. Overexpressed DjlA, along with DnaK, may alter the conformation of the inner membrane components involved in the Rcs pathway, potentially disrupting the inhibitory action of IgaA on RcsD [86]. As mentioned earlier, BipA exhibits chaperone-like activity and plays a role in the LPS core biosynthesis, which primarily occurs at the inner leaflet of the cytoplasmic membrane. This leads us to hypothesize that BipA may influence a specific conformation involving the LPS core-synthesizing enzymes and/or Rcs components. Further investigations are required to identify which RcsF-independent signals arise in the *bipA*-deleted strain and how they contribute to signaling activation.

In *S*. Typhimurium, overexpression of RcsA, a defective mutant IgaA, or constitutively activated RcsC leads to colanic acid overproduction, even at 37°C, resulting in mucoid colony morphology and loss of motility [65,87,88]. Although the Rcs pathway is highly conserved between *E. coli* and *S*. Typhimurium, our finding that capsule synthesis was unaffected by the *bipA* deletion in *S*. Typhimurium strains is particularly intriguing. Notably, there are differences in the lipid A-core structure between the two species [56,89], and as shown in Fig 5A, wild-type *E. coli* and *S*. Typhimurium strains exhibited maximum resistance to bile salts at concentrations of 2^6^ g/L and 2^5^ g/L at 37°C and 20°C, respectively. Additionally, *E. coli* cells were more sensitive to bile salts than *S*. Typhimurium cells at low temperatures. This indicates that the defective LPS in the *bipA*-deleted strain may cause varying degrees of damage to the cell envelope. Furthermore, the formation of the RcsB-RcsA heterodimer, essential for activating the *cps* gene cluster, is regulated at multiple levels. In normally growing cells, RcsA is limiting for *cps* gene transcription because H-NS represses *rcsA* transcription [90]. This repression is alleviated by a small, 85-nucleotide RNA molecule, DsrA [91]. Additionally, the quantity of RcsA protein is tightly regulated by the Lon protease [60]. Consequently, the LPS defects in the *bipA*-deleted *Salmonella* strains may not produce sufficient RcsB-RcsA heterodimers formation to induce sufficient *cps* gene cluster expression (S5C Fig). However, we do not rule out the possibility of alternative regulatory mechanisms affecting capsular polysaccharide production in *S*. Typhimurium.

The deletion of *bipA* did not uniformly induce mucoid colony morphology in all four strains, leading to varied impacts on biofilm formation among the four strains. Biofilm formation disruption due to *bipA* deletion was observed only in the MG1655 and 14028 strains, while in the SL1344 and LT2 strains (Fig 6). *S*. Typhimurium primarily produces cellulose as the main exopolysaccharide compound for biofilm matrix formation [92]. RpoS positively regulates *mlrA*, whose gene product subsequently upregulates CsgD, a key player in curli synthesis. CsgD also stimulates the expression of AdrA, which regulates cellulose production. However, in the SL1344 and LT2 strains, the transcription of *mlrA* and the levels of RpoS were either defective or nearly undetectable, respectively [34,93–95]. These discrepancies in the expression of positive regulators resulted in the cellulose-deficient phenotype, explaining the lack of inherent biofilm formation in the SL1344 and LT2 strains under the tested conditions.

Unlike the *cps* gene, which regulates colanic acid production through the activation of the RcsB-RcsA heterodimer, this complex inhibits flagellum synthesis by repressing *flhDC* within the Rcs pathway. Notably, *bipA*-deleted *Salmonella* strains exhibited only a moderate motility defect despite having a similar number of flagella at 37°C. Additionally, qRT-PCR and western blot analyses revealed no significant changes in the expression levels of flagellum structural genes and FliC proteins at 37°C (S6 and S7 Figs). However, Δ*bipA* cells exhibited an increase in the expression of type 1 fimbriae at 37°C (S9 Fig), whose overexpression partially reduced flagella-mediated motility [96]. Furthermore, this motility defect at 37°C may be attributed to the reduced ATP levels observed in all *bipA*-deleted strains (S10 Fig). Apparently, a significant motility defect was observed in the *bipA*-deleted SL1344 and 14028 strains, with a clear reduction in flagella count at 20°C (Fig 7). In the MG1655 and LT2 strains, although the transcriptional expression of flagellum-related genes significantly reduced in the corresponding mutant strains compared to the wild-type strain at 20°C (S7 Fig), this did not result in a reduced flagella count, as these strains inherently have fewer flagella. Nevertheless, the mutant MG1655 and LT2 strains showed impaired motility at 20°C.

Overall, our findings indicate that BipA is involved not only in flagellum biosynthesis but also in the quality and functionality of the flagellum. Notably, EptC, a phosphoethanolamine transferase, plays a dual role in modifying the flagellum rod protein FlgG, and the lipid A substructure of *Campylobacter jejuni* LPS with a phosphoethanolamine residue. The *eptC*-deficient stains exhibited decreased motility and flagella formation [97,98]. This suggests that the defects in motility and flagella count observed in the *bipA* mutant may result from the combined effects of the Rcs pathway and the absence of interaction of BipA with LPS-modifying or -synthesizing enzymes. Further studies are needed to elucidate the exact molecular mechanisms by which BipA influences these processes and to clarify its potential role in coordinating flagella formation and LPS biosynthesis.

LPS, capsules, biofilms, and flagella are critical virulence determinants, along with T3SSs and the bacterial energy state, both of which are crucial for virulence. The pronounced defects in motility observed in the pathogenic *S*. Typhimurium SL1344 and 14028 strains at 37°C highlight the essential role of BipA in pathogenicity (Fig 8). Moreover, our data indicate that deletion of *bipA* leads to a significant reduction in the expression of T3SS genes only at lower temperatures (S11 Fig), suggesting that the observed virulence attenuation is not due to T3SS repression. Specifically, decreased motility and ATP levels in *bipA*-deleted strains (Figs 7 and S10) suggest that BipA contributes to energy homeostasis, which may, in turn, impact virulence-related functions in host infection models (Fig 8).

In addition, BipA is important for bacterial resistance to host antimicrobial mechanisms. Given that *Salmonella* encounters ROS during infection, it must resist oxidative stress to survive. The *bipA*-deleted strains exhibited increased susceptibility to H_2_O_2_ at both 37°C and 20°C (S12 Fig), suggesting that BipA may contribute to bacterial defense mechanisms against oxidative damage in host cells.

Thus, our findings indicate that BipA functions as a key fitness factor, modulating surface-associated structures, including flagella, LPS, and T3SS, and plays a crucial role in maintaining bacterial energy balance and enhancing resistance to oxidative stress. Future research aimed at elucidating the mechanisms by which BipA regulates membrane integrity, responses to various stresses, and virulence will provide insights into bacterial adaptation and pathogenesis.

## Supporting information

S1 Supporting Information Supplementary Materials and Methods. This section provides detailed descriptions of the experimental procedures, including cell morphology observation, the swarming motility assay, the deflagellation method, growth curve measurement, the Epsilometer test, and the ATP assay.
(DOCX)

S1 Fig Normal growth of *bipA*-deleted *E. coli* and *S*. Typhimurium at 37°C. The growth of wild-type and *bipA*-deleted strains of *E. coli* and *S*. Typhimurium was assessed at 37°C. (**A**) Overnight cultures were diluted and spotted, as described in Fig 1A, followed by incubation at 37°C. (**B** and **C**) Cells grown at 37°C to an OD_600_ of 0.5 were inoculated into fresh LB medium and further incubated at 37°C for 12 h. Error bars indicate SD. (**D**) Conserved CRP-binding site upstream of the *SebipA* promoter. The upstream DNA sequences of *E. coli* and *S*. Typhimurium are aligned, with the consensus CRP-binding site highlighted in yellow, the −35 and −10 regions in blue, and the initiation codon GTG in green.
(TIF)

S2 Fig Sequence alignment of the BipA proteins. (**A**) Sequence alignment of BipA from *E. coli* and *S*. Typhimurium was conducted using ClustalW and ESPript. The numbers indicate the corresponding residues, and the blue arrowhead indicates the Asn128 residue. (BipA, GenBank accession number: AAT48232; SeBipA, CAC14270). (**B**) Expression of wild-type and mutant BipA proteins in MG1655Δ*bipA* and mutant *S*. Typhimurium cells. The transformants shown in Fig 2B underwent Western blot analysis using an anti-BipA antibody. M: PageRuler Prestained Protein Ladder (Thermo Fisher Scientific). (**C**) Colony formation of cross-complemented *bipA*-deletion strains at 37°C. The *bipA*-deleted *E. coli* and *S*. Typhimurium strains were transformed with the plasmid as shown. Transformants were spotted as described in Fig 2B, and the plates were incubated at 37°C.
(TIF)

S3 Fig Multiple sequence alignment analysis of the intervening sequences (IVSs) in the 23S rRNA genes. The nucleotide sequences of the 23S rRNA genes from *E. coli* MG1655 (**A** and **B**) and *S*. Typhimurium SL1344 (**C** and **D**), 14028 (**E** and **F**), and LT2 (**G** and **H**) were analyzed. The first IVSs in the helix 25 region (**A**, **C**, **E**, and **G**) and the second IVSs in the helix 45 region (**B**, **D**, **F**, and **H**) were aligned using Clustal Omega [99] and visualized using Jalview [100].
(TIF)

S4 Fig Analysis of LPS in *E. coli* and *S.* Typhimurium strains. (**A**) LPS profile analysis of the *E. coli* and *S*. Typhimurium strains. LPS was extracted and analyzed, as described in Fig 5. **(B)** Colony morphology of *bipA*, *rcsF*, *rcsA*, and *lon* mutants. Overnight cultures were diluted to an OD_600_ of 0.04 in the LB medium and then further diluted 10^−3^-fold. One hundred microliters of the diluted culture were spread with glass beads on LB agar plates and incubated at 37°C or 20°C.
(TIF)

S5 Fig Effect of *bipA* deletion on capsule production. (**A**) Colony morphology of wild-type and *bipA*-deleted strains of *E. coli* and *S*. Typhimurium. Overnight cultures were diluted and spread as in S4B Fig. (**B**) Macrocolony assay of the same strains at 37°C for 18 h or 20°C for 7 d. Overnight cultures were diluted to an OD_600_ of 0.02 in the same medium, and 3 μL of the diluted cultures were spotted on MacConkey agar plates. **(C)** Relative quantification of g*md* mRNA levels in the *cps* cluster. Total RNA was extracted from cells grown to an OD_600_ of 0.5 and analyzed using qRT-PCR. The relative quantity was normalized to the *rrsA* gene and expressed relative to the transcript level of the wild-type strain grown at 37°C.
(TIF)

S6 Fig Reduced swarming motility and flagellin production in *bipA*-deleted *S.* Typhimurium strains at low temperatures. (**A**) Swarming motility assay of wild-type and *bipA*-deleted strains of *E. coli* and *S*. Typhimurium at 37°C or 20°C. The cells were grown and diluted, as described in Fig 7A. The cultures were concentrated 10-fold and spotted on an LB agar plate containing glucose. Plates were incubated at 37°C for 14 h or 20°C for 2 d. (**B**) Reduced flagellin production in the Δ*bipA* of *S*. Typhimurium at 20°C. Overnight cultures were diluted 10^−2^-fold in fresh medium and incubated until the OD_600_ reached 0.5 at 37°C or 20°C. Deflagellation was performed as described in the Materials and Methods section. Western blot analysis of the wild-type and *bipA*-deleted strain samples was conducted using an anti-FliC antibody. To determine the locations of FliC and FljB, the lysates of MG1655 cells harboring pIN-A, pINFliC, pINSeFliC, or pINSeFljB were loaded onto SDS-PAGE.
(TIF)

S7 Fig Repression of flagella-related gene transcription in *bipA*-deleted strains at low temperatures. Total RNA was extracted from the wild-type and *bipA*-deleted strains cultured in the early exponential phase at 37°C or 20°C and analyzed using qRT-PCR. The relative expression levels of *flgM*, *fliE*, *fliK*, *fliM*, *fliS*, and *fliZ* were normalized to the endogenous control gene *rrsA*.
(TIF)

S8 Fig Increased polymyxin B susceptibility in *bipA*-deleted strains at 20°C. **(A)** Growth curves of wild-type and *bipA*-deleted strains in the presence of polymyxin B. Overnight cultures were diluted 200-fold in fresh LB medium. A 180 μL aliquot of the diluted culture was added to each well of a 96-well plate, along with 20 μL of polymyxin B or distilled water as a control. Plates were incubated at 37°C for 18 h or at 20°C for 48 h with shaking. **(B)** ΔLag time between wild-type and *bipA*-deleted strains. Lag time was determined using Gen5 software (Agilent Technologies). ΔLag time was calculated as the lag time of the mutant strain minus the lag time of the wild-type strain. **(C)** Polymyxin B E-test for MIC determination. A polymyxin B MIC test strip was placed onto LB agar plates spread with diluted cultures of wild-type or *bipA*-deleted strains, followed by incubation at 37°C or 20°C.
(TIF)

S9 Fig Elevated expression of *fimD* in *bipA*-deleted strains. Total RNA was extracted from wild-type and *bipA*-deleted strains in the early exponential phase at 37°C or 20°C and analyzed using qRT-PCR. The relative expression levels of the type 1 fimbriae gene *fimD* were normalized to the endogenous control gene *rrsA*.
(TIF)

S10 Fig ATP levels in wild-type and *bipA*-deleted strains at 37°C and 20°C. Lysate samples were prepared from wild-type and *bipA*-deleted strains cultured in the early exponential phase at 37°C or 20°C, as described in S1 Supporting Information. A 50 μL sample was mixed with 50 μL of luciferase reagent, followed by luminescence measurement. ATP concentrations were calculated based on a standard curve ranging from 10^-5^-10^-10^ M.
(TIF)

S11 Fig Decreased expression levels of SPI-1 genes in *bipA*-deleted strain at 20°C. Total RNA was extracted from the wild-type and *bipA*-deleted cells incubated at 37°C or 20°C to the early exponential phase and analyzed using qRT-PCR. The relative expression levels of *hilA*, *invA*, *prgH*, and *sipA* were normalized to the endogenous control gene *rrsA*.
(TIF)

S12 Fig BipA is required for optimal growth in the presence of ROS. **(A)** Growth curves of wild-type and *bipA*-deleted strains of *E. coli* and *S*. Typhimurium in the presence of H_2_O_2_. Growth was measured as described in S8A Fig. **(B)** Increased lag time in *bipA*-deleted strains in the presence of H_2_O_2_. ΔLag time was calculated as described in S8B Fig.
(TIF)

S1 Table Primers used in this study. List of primers used for strains and plasmids construction, and qRT-PCR.
(DOCX)

S1 Data Source data for graphs in this study. This file contains the raw data used to generate all the graphs presented in the manuscript, as well as the data points extracted from images used in the analysis.
(XLSX)

## References

[1] Bourne HR, Sanders DA, McCormick F. The GTPase superfamily: conserved structure and molecular mechanism. Nature. 1991;349(6305):117–27. doi: 10.1038/349117a0 1898771

[2] Leipe DD, Wolf YI, Koonin EV, Aravind L. Classification and evolution of P-loop GTPases and related ATPases. J Mol Biol. 2002;317(1):41–72. doi: 10.1006/jmbi.2001.5378 11916378

[3] Ero R, Kumar V, Chen Y, Gao Y-G. Similarity and diversity of translational GTPase factors EF-G, EF4, and BipA: From structure to function. RNA Biol. 2016;13(12):1258–73. doi: 10.1080/15476286.2016.1201627 27325008 PMC5207388

[4] Fan H, Hahm J, Diggs S, Perry JJP, Blaha G. Structural and functional analysis of BipA, a regulator of virulence in enteropathogenic *Escherichia coli*. J Biol Chem. 2015;290(34):20856–64. doi: 10.1074/jbc.M115.659136 26163516 PMC4543647

[5] Qi S, Li Y, Szyroki A, Giles I, Moir A, Oconnor C. *Salmonella typhimurium* responses to a bactericidal protein from human neutrophils. Mol Microbiol. 1995;17(3):523–31. 8559071 10.1111/j.1365-2958.1995.mmi_17030523.x

[6] Dallegri F, Ottonello L. Tissue injury in neutrophilic inflammation. Inflamm Res. 1997;46(10):382–91. doi: 10.1007/s000110050208 9372309

[7] Bainton DF. Neutrophilic leukocyte granules: from structure to function. Adv Exp Med Biol. 1993;336:17–33. doi: 10.1007/978-1-4757-9182-2_3 8296606

[8] Mayadas T, Cullere X, Lowell C. The multifaceted functions of neutrophils. Annu Rev Pathol. 2014;9:181–218. 24050624 10.1146/annurev-pathol-020712-164023 PMC4277181

[9] Weiss J, Elsbach P, Olsson I, Odeberg H. Purification and characterization of a potent bactericidal and membrane active protein from the granules of human polymorphonuclear leukocytes. J Biol Chem. 1978;253(8):2664–72. doi: 10.1016/s0021-9258(17)40872-6 344320

[10] Ooi CE, Weiss J, Elsbach P, Frangione B, Mannion B. A 25-kDa NH_2_-terminal fragment carries all the antibacterial activities of the human neutrophil 60-kDa bactericidal/permeability-increasing protein. J Biol Chem. 1987;262(31):14891–4. doi: 10.1016/s0021-9258(18)48110-0 3667613

[11] Schumann RR, Leong SR, Flaggs GW, Gray PW, Wright SD, Mathison JC, et al. Structure and function of lipopolysaccharide binding protein. Science. 1990;249(4975):1429–31. doi: 10.1126/science.2402637 2402637

[12] Kirschning CJ, Au-Young J, Lamping N, Reuter D, Pfeil D, Seilhamer JJ, et al. Similar organization of the lipopolysaccharide-binding protein (LBP) and phospholipid transfer protein (PLTP) genes suggests a common gene family of lipid-binding proteins. Genomics. 1997;46(3):416–25. doi: 10.1006/geno.1997.5030 9441745

[13] Gazzano-Santoro H, Parent JB, Grinna L, Horwitz A, Parsons T, Theofan G, et al. High-affinity binding of the bactericidal/permeability-increasing protein and a recombinant amino-terminal fragment to the lipid A region of lipopolysaccharide. Infect Immun. 1992;60(11):4754–61. doi: 10.1128/iai.60.11.4754-4761.1992 1398985 PMC258228

[14] Vaara M. Agents that increase the permeability of the outer-membrane. Microbiol Rev. 1992;56(3):395-411. 1406489 10.1128/mr.56.3.395-411.1992 PMC372877

[15] Ebbensgaard A, Mordhorst H, Aarestrup F, Hansen E. The role of outer membrane proteins and lipopolysaccharides for the sensitivity of *Escherichia coli* to antimicrobial peptides. Front Microbiol. 2018;9:2153. 30245684 10.3389/fmicb.2018.02153 PMC6137088

[16] Mannion B, Kalatzis E, Weiss J, Elsbach P. Preferential binding of the neutrophil cytoplasmic granule-derived bactericidal/permeability increasing protein to target bacteria. Implications and use as a means of purification. J Immunol. 1989;142(8):2807–12. 2539411

[17] Elsbach P. Bactericidal permeability-increasing protein in host defence against Gram-negative bacteria and endotoxin. Ciba Found Symp. 1994;186:176–87; discussion 187-9. doi: 10.1002/9780470514658.ch11 7768151

[18] Elsbach P. The bactericidal/permeability-increasing protein (BPI) in antibacterial host defense. J Leukoc Biol. 1998;64(1):14–8. 9665269 10.1002/jlb.64.1.14

[19] Iovine N, Elsbach P, Weiss J. An opsonic function of the neutrophil bactericidal permeability-increasing protein depends on both its N- and C-terminal domains. Proc Natl Acad Sci U S A. 1997;94(20):10973–8. 9380744 10.1073/pnas.94.20.10973 PMC23549

[20] Groisman EA, Parra-Lopez C, Salcedo M, Lipps CJ, Heffron F. Resistance to host antimicrobial peptides is necessary for *Salmonella* virulence. Proc Natl Acad Sci U S A. 1992;89(24):11939–43. doi: 10.1073/pnas.89.24.11939 1465423 PMC50673

[21] Weiss J, Muello K, Victor M, Elsbach P. The role of lipopolysaccharides in the action of the bactericidal/permeability-increasing neutrophil protein on the bacterial envelope. J Immunol. 1984;132(6):3109–15. doi: 10.4049/jimmunol.132.6.3109 6373924

[22] Weiss J, Hutzler M, Kao L. Environmental modulation of lipopolysaccharide chain length alters the sensitivity of *Escherichia coli* to the neutrophil bactericidal/permeability-increasing protein. Infect Immun. 1986;51(2):594–9. doi: 10.1128/iai.51.2.594-599.1986 3510983 PMC262384

[23] Weiss J, Beckerdite-Quagliata S, Elsbach P. Resistance of Gram-negative bacteria to purified bactericidal leukocyte proteins: relation to binding and bacterial lipopolysaccharide structure. J Clin Invest. 1980;65(3):619–28. doi: 10.1172/JCI109707 6986410 PMC371403

[24] Capodici C, Chen S, Sidorczyk Z, Elsbach P, Weiss J. Effect of lipopolysaccharide (LPS) chain length on interactions of bactericidal/permeability-increasing protein and its bioactive 23-kilodalton NH_2_-terminal fragment with isolated LPS and intact *Proteus mirabilis* and *Escherichia coli*. Infect Immun. 1994;62(1):259–65. 8262637 10.1128/iai.62.1.259-265.1994 PMC186095

[25] Farris M, Grant A, Richardson TB, O’Connor CD. BipA: a tyrosine-phosphorylated GTPase that mediates interactions between enteropathogenic *Escherichia coli* (EPEC) and epithelial cells. Mol Microbiol. 1998;28(2):265–79. doi: 10.1046/j.1365-2958.1998.00793.x 9622352

[26] Crane SD, Banerjee SK, Eichelberger KR, Kurten RC, Goldman WE, Pechous RD. The *Yersinia pestis* GTPase BipA promotes pathogenesis of primary pneumonic plague. Infect Immun. 2021;89(2):e00673-20. doi: 10.1128/IAI.00673-20 33257531 PMC7822129

[27] Choi E, Hwang J. The GTPase BipA expressed at low temperature in *Escherichia coli* assists ribosome assembly and has chaperone-like activity. J Biol Chem. 2018;293(47):18404–19. 30305394 10.1074/jbc.RA118.002295 PMC6254333

[28] Choi E, Jeon H, Oh C, Hwang J. Elucidation of a novel role of YebC in surface polysaccharides regulation of *Escherichia coli bipA*-deletion. Front Microbiol. 2020;11:597515. 33240252 10.3389/fmicb.2020.597515 PMC7682190

[29] Neidig A, Yeung ATY, Rosay T, Tettmann B, Strempel N, Rueger M, et al. TypA is involved in virulence, antimicrobial resistance and biofilm formation in *Pseudomonas aeruginosa*. BMC Microbiol. 2013;13:77. doi: 10.1186/1471-2180-13-77 23570569 PMC3639842

[30] Datsenko KA, Wanner BL. One-step inactivation of chromosomal genes in *Escherichia coli* K-12 using PCR products. Proc Natl Acad Sci U S A. 2000;97(12):6640–5. doi: 10.1073/pnas.120163297 10829079 PMC18686

[31] Blattner FR, Plunkett G 3rd, Bloch CA, Perna NT, Burland V, Riley M, et al. The complete genome sequence of *Escherichia coli* K-12. Science. 1997;277(5331):1453–62. doi: 10.1126/science.277.5331.1453 9278503

[32] Baba T, Ara T, Hasegawa M, Takai Y, Okumura Y, Baba M. Construction of *Escherichia coli* K-12 in-frame, single-gene knockout mutants: the Keio collection. Mol Syst Biol. 2006;2:2006–0008. doi: 10.1038/msb.2006.8 PMC1681482 16738554

[33] Hoiseth SK, Stocker BA. Aromatic-dependent *Salmonella typhimurium* are non-virulent and effective as live vaccines. Nature. 1981;291(5812):238–9. doi: 10.1038/291238a0 7015147

[34] Swords WE, Cannon BM, Benjamin WH. A virulence of LT2 strains of *Salmonella typhimurium* results from a defective *rpoS* gene. Infect Immun. 1997;65(6):2451–3. 9169789 10.1128/iai.65.6.2451-2453.1997 PMC175341

[35] Ghrayeb J, Kimura H, Takahara M, Hsiung H, Masui Y, Inouye M. Secretion cloning vectors in *Escherichia coli*. EMBO J. 1984;3(10):2437–42. doi: 10.1002/j.1460-2075.1984.tb02151.x 6094184 PMC557705

[36] Sarmientos P, Sylvester JE, Contente S, Cashel M. Differential stringent control of the tandem *E. coli* ribosomal RNA promoters from the *rrnA* operon expressed *in vivo* in multicopy plasmids. Cell. 1983;32(4):1337–46. doi: 10.1016/0092-8674(83)90314-8 6188537

[37] Livak KJ, Schmittgen TD. Analysis of relative gene expression data using real-time quantitative PCR and the 2^-^^ΔΔ^^*C*T^ Method. Methods. 2001;25(4):402–8. doi: 10.1006/meth.2001.1262 11846609

[38] Kenyon JJ, Reeves PR. The Wzy O-antigen polymerase of *Yersinia pseudotuberculosis* O:2a has a dependence on the Wzz chain-length determinant for efficient polymerization. FEMS Microbiol Lett. 2013;349(2):163–70. doi: 10.1111/1574-6968.12311 24164168

[39] Morrissey JH. Silver stain for proteins in polyacrylamide gels: a modified procedure with enhanced uniform sensitivity. Anal Biochem. 1981;117(2):307–10. doi: 10.1016/0003-2697(81)90783-1 6172996

[40] Han S, Byun J, Lee M. Comparative transcriptomic analysis of flagellar-associated genes in *Salmonella* Typhimurium and its *rnc* mutant. J Microbiol. 2024;62(1):33–48. 38182942 10.1007/s12275-023-00099-5

[41] Lee M, Ryu M, Joo M, Seo Y-J, Lee J, Kim H-M, et al. Endoribonuclease-mediated control of *hns* mRNA stability constitutes a key regulatory pathway for *Salmonella* Typhimurium pathogenicity island 1 expression. PLoS Pathog. 2021;17(2):e1009263. doi: 10.1371/journal.ppat.1009263 33524062 PMC7877770

[42] Mantel N. Evaluation of survival data and two new rank order statistics arising in its consideration. Cancer Chemother Rep. 1966;50(3):163–70. 5910392

[43] Brandi A, Spurio R, Gualerzi CO, Pon CL. Massive presence of the *Escherichia coli* “major cold-shock protein” CspA under non-stress conditions. EMBO J. 1999;18(6):1653–9. doi: 10.1093/emboj/18.6.1653 10075935 PMC1171252

[44] Burgin AB, Parodos K, Lane DJ, Pace NR. The excision of intervening sequences from *Salmonella* 23S ribosomal RNA. Cell. 1990;60(3):405–14. doi: 10.1016/0092-8674(90)90592-3 2406020

[45] Evguenieva-Hackenberg E. Bacterial ribosomal RNA in pieces. Mol Microbiol. 2005;57(2):318–25. doi: 10.1111/j.1365-2958.2005.04662.x 15978067

[46] deLivron MA, Robinson VL. *Salmonella enterica* serovar Typhimurium BipA exhibits two distinct ribosome binding modes. J Bacteriol. 2008;190(17):5944–52. doi: 10.1128/JB.00763-08 18621905 PMC2519513

[47] Ren G, Wang Z, Li Y, Hu X, Wang X. Effects of lipopolysaccharide core sugar deficiency on colanic acid biosynthesis in *Escherichia coli*. J Bacteriol. 2016;198(11):1576–84. 27002133 10.1128/JB.00094-16 PMC4959291

[48] Majdalani N, Gottesman S. The Rcs phosphorelay: a complex signal transduction system. Annu Rev Microbiol. 2005;59:379–405. doi: 10.1146/annurev.micro.59.050405.101230 16153174

[49] Ebel W, Trempy JE. *Escherichia coli* RcsA, a positive activator of colanic acid capsular polysaccharide synthesis, functions to activate its own expression. J Bacteriol. 1999;181(2):577–84. doi: 10.1128/JB.181.2.577-584.1999 9882673 PMC93413

[50] Francez-Charlot A, Laugel B, Van Gemert A, Dubarry N, Wiorowski F, Castanié-Cornet M-P, et al. RcsCDB His-Asp phosphorelay system negatively regulates the *flhDC* operon in *Escherichia coli*. Mol Microbiol. 2003;49(3):823–32. doi: 10.1046/j.1365-2958.2003.03601.x 12864862

[51] Hu LI, Chi BK, Kuhn ML, Filippova EV, Walker-Peddakotla AJ, Bäsell K, et al. Acetylation of the response regulator RcsB controls transcription from a small RNA promoter. J Bacteriol. 2013;195(18):4174–86. doi: 10.1128/JB.00383-13 23852870 PMC3754749

[52] Linkevicius M, Anderssen JM, Sandegren L, Andersson DI. Fitness of *Escherichia coli* mutants with reduced susceptibility to tigecycline. J Antimicrob Chemother. 2016;71(5):1307–13. doi: 10.1093/jac/dkv486 26851608 PMC4830415

[53] May JF, Groisman EA. Conflicting roles for a cell surface modification in *Salmonella*. Mol Microbiol. 2013;88(5):970–83. doi: 10.1111/mmi.12236 23646936 PMC4081025

[54] Santos LGA-A, Musther H, Bala N, Deferm N, Patel G, Brouwers J, et al. Gastrointestinal bile salt concentrations in healthy adults under fasted and fed conditions: a systematic review and meta-analysis for mechanistic physiologically-based pharmacokinetic (PBPK) modelling. AAPS J. 2025;27(1):31. doi: 10.1208/s12248-025-01016-x 39843813

[55] Gronow S, Brabetz W, Brade H. Comparative functional characterization *in vitro* of heptosyltransferase I (WaaC) and II (WaaF) from *Escherichia coli*. Eur J Biochem. 2000;267(22):6602–11. 11054112 10.1046/j.1432-1327.2000.01754.x

[56] Qian J, Garrett TA, Raetz CRH. *In vitro* assembly of the outer core of the lipopolysaccharide from *Escherichia coli* K-12 and *Salmonella typhimurium*. Biochemistry. 2014;53(8):1250–62. doi: 10.1021/bi4015665 24479701 PMC3985525

[57] Mudapaka J, Taylor EA. Cloning and characterization of the *Escherichia coli* Heptosyltransferase III: Exploring substrate specificity in lipopolysaccharide core biosynthesis. FEBS Lett. 2015;589(13):1423–9. doi: 10.1016/j.febslet.2015.04.051 25957775

[58] Henriksen SD. Studies in mucoid *Escherichia coli* II. specificity of the mucoid antigen (M‐Antigen). APMIS. 1949;26(6):903–13. doi: 10.1111/j.1699-0463.1949.tb00794.x

[59] Meredith TC, Mamat U, Kaczynski Z, Lindner B, Holst O, Woodard RW. Modification of lipopolysaccharide with colanic acid (M-antigen) repeats in *Escherichia coli*. J Biol Chem. 2007;282(11):7790–8. doi: 10.1074/jbc.M611034200 17227761

[60] Stout V, Torres-Cabassa A, Maurizi M, Gutnick D, Gottesman S. RcsA, an unstable positive regulator of capsular polysaccharide synthesis. J Bacteriol. 1991;173(5):1738–47. 1999391 10.1128/jb.173.5.1738-1747.1991 PMC207325

[61] Marolda CL, Vicarioli J, Valvano MA. Wzx proteins involved in biosynthesis of O antigen function in association with the first sugar of the O-specific lipopolysaccharide subunit. Microbiology (Reading). 2004;150(Pt 12):4095–105. doi: 10.1099/mic.0.27456-0 15583162

[62] Bishop RE, Gibbons HS, Guina T, Trent MS, Miller SI, Raetz CR. Transfer of palmitate from phospholipids to lipid A in outer membranes of Gram-negative bacteria. EMBO J. 2000;19(19):5071–80. doi: 10.1093/emboj/19.19.5071 11013210 PMC302101

[63] Garegg J, Lindberg B, Onn T, Holme T. Structural studies on the M-antigen from two mucoid mutants of *Salmonella typhimurium*. Acta Chem Scand. 1971;25(4):1185–94. doi: 10.3891/acta.chem.scand.25-1185 4939458

[64] Hellerqvist CG, Larm O, Lindberg B. Structure of an oligosaccharide obtained on degradation of the lipopolysaccharide from *Salmonella typhimurium* LT2. Acta Chem Scand. 1971;25(2):744–5. doi: 10.3891/acta.chem.scand.25-0744 4936224

[65] Pando JM, Karlinsey JE, Lara JC, Libby SJ, Fang FC. The Rcs-regulated colanic acid capsule maintains membrane potential in *Salmonella enterica* serovar Typhimurium. mBio. 2017;8(3):e00808-17. doi: 10.1128/mBio.00808-17 28588134 PMC5461412

[66] Majdalani N, Hernandez D, Gottesman S. Regulation and mode of action of the second small RNA activator of RpoS translation, RprA. Mol Microbiol. 2002;46(3):813–26. 12410838 10.1046/j.1365-2958.2002.03203.x

[67] Jørgensen MG, Nielsen JS, Boysen A, Franch T, Møller-Jensen J, Valentin-Hansen P. Small regulatory RNAs control the multi-cellular adhesive lifestyle of *Escherichia coli*. Mol Microbiol. 2012;84(1):36–50. doi: 10.1111/j.1365-2958.2012.07976.x 22250746

[68] Ogasawara H, Yamamoto K, Ishihama A. Role of the biofilm master regulator CsgD in cross-regulation between biofilm formation and flagellar synthesis. J Bacteriol. 2011;193(10):2587–97. doi: 10.1128/JB.01468-10 21421764 PMC3133154

[69] Roberts IS. The biochemistry and genetics of capsular polysaccharide production in bacteria. Annu Rev Microbiol. 1996;50:285–315. doi: 10.1146/annurev.micro.50.1.285 8905082

[70] Chitlapilly Dass S, Wang R. Biofilm through the looking glass: a microbial food safety perspective. Pathogens. 2022;11(3):346. doi: 10.3390/pathogens11030346 35335670 PMC8954374

[71] Tan MSF, White AP, Rahman S, Dykes GA. Role of fimbriae, flagella and cellulose on the attachment of *Salmonella* Typhimurium ATCC 14028 to plant cell wall models. PLoS One. 2016;11(6):e0158311. doi: 10.1371/journal.pone.0158311 27355584 PMC4927157

[72] Xu H, Zou Y, Lee H-Y, Ahn J. Effect of NaCl on the biofilm formation by foodborne pathogens. J Food Sci. 2010;75(9):M580-5. doi: 10.1111/j.1750-3841.2010.01865.x 21535614

[73] Duan Q, Zhou M, Zhu L, Zhu G. Flagella and bacterial pathogenicity. J Basic Microbiol. 2013;53(1):1–8. doi: 10.1002/jobm.201100335 22359233

[74] Echazarreta MA, Klose KE. *Vibrio* flagellar synthesis. Front Cell Infect Microbiol. 2019;9:131. 31119103 10.3389/fcimb.2019.00131 PMC6504787

[75] Lee J, Shin E, Yeom J-H, Park J, Kim S, Lee M, et al. Regulator of RNase E activity modulates the pathogenicity of *Salmonella* Typhimurium. Microb Pathog. 2022;165:105460. doi: 10.1016/j.micpath.2022.105460 35231570

[76] Xue S, Barna M. Specialized ribosomes: a new frontier in gene regulation and organismal biology. Nat Rev Mol Cell Biol. 2012;13(6):355–69. doi: 10.1038/nrm3359 22617470 PMC4039366

[77] Colon-Ramos D, Shenvi C, Weitzel D, Gan E, Matts R, Cate J. Direct ribosomal binding by a cellular inhibitor of translation. Nat Struct Mol Biol. 2006;13(2):103–11. 16429152 10.1038/nsmb1052 PMC2741086

[78] Baum S, Bittins M, Frey S, Seedorf M. Asc1p, a WD40-domain containing adaptor protein, is required for the interaction of the RNA-binding protein Scp160p with polysomes. Biochem J. 2004;380(Pt 3):823–30. doi: 10.1042/BJ20031962 15012629 PMC1224212

[79] Coyle SM, Gilbert WV, Doudna JA. Direct link between RACK1 function and localization at the ribosome *in vivo*. Mol Cell Biol. 2009;29(6):1626–34. doi: 10.1128/MCB.01718-08 19114558 PMC2648249

[80] Li A-M, Watson A, Fridovich-Keil JL. Scp160p associates with specific mRNAs in yeast. Nucleic Acids Res. 2003;31(7):1830–7. doi: 10.1093/nar/gkg284 12654998 PMC152800

[81] Simpson BW, Trent MS. Pushing the envelope: LPS modifications and their consequences. Nat Rev Microbiol. 2019;17(7):403–16. doi: 10.1038/s41579-019-0201-x 31142822 PMC6913091

[82] Klein G, Raina S. Regulated assembly of LPS, its structural alterations and cellular response to LPS defects. Int J Mol Sci. 2019;20(2). doi: 10.3390/ijms20020356 PMC6358824 30654491

[83] Stokes JM, French S, Ovchinnikova OG, Bouwman C, Whitfield C, Brown ED. Cold stress makes *E**scherichia coli* susceptible to glycopeptide antibiotics by altering outer membrane integrity. Cell Chem Biol. 2016;23(2):267–77. doi: 10.1016/j.chembiol.2015.12.011 26853624

[84] Klein G, Lindner B, Brade H, Raina S. Molecular basis of lipopolysaccharide heterogeneity in *Escherichia coli*: envelope stress-responsive regulators control the incorporation of glycoforms with a third 3-deoxy-α-D-*manno*-oct-2-ulosonic acid and rhamnose. J Biol Chem. 2011;286(50):42787–807. doi: 10.1074/jbc.M111.291799 22021036 PMC3234843

[85] Meng J, Young G, Chen J. The Rcs system in *E**nterobacteriaceae*: envelope stress responses and virulence regulation. Front Microbiol. 2021;12:627104. doi: 10.3389/fmicb.2021.627104 33658986 PMC7917084

[86] Shiba Y, Yokoyama Y, Aono Y, Kiuchi T, Kusaka J, Matsumoto K, et al. Activation of the Rcs signal transduction system is responsible for the thermosensitive growth defect of an *Escherichia coli* mutant lacking phosphatidylglycerol and cardiolipin. J Bacteriol. 2004;186(19):6526–35. doi: 10.1128/JB.186.19.6526-6535.2004 15375134 PMC516613

[87] Pucciarelli MG, Rodríguez L, García-Del Portillo F. A disulfide bond in the membrane protein IgaA is essential for repression of the RcsCDB system. Front Microbiol. 2017;8:2605. doi: 10.3389/fmicb.2017.02605 29312270 PMC5744062

[88] García-Calderón CB, García-Quintanilla M, Casadesús J, Ramos-Morales F. Virulence attenuation in *Salmonella enterica rcsC* mutants with constitutive activation of the Rcs system. Microbiology (Reading). 2005;151(Pt 2):579–88. doi: 10.1099/mic.0.27520-0 15699206

[89] Zhou Z, Lin S, Cotter RJ, Raetz CR. Lipid A modifications characteristic of *Salmonella typhimurium* are induced by NH_4_VO_3_ in *Escherichia coli* K12. Detection of 4-amino-4-deoxy-L-arabinose, phosphoethanolamine and palmitate. J Biol Chem. 1999;274(26):18503–14. doi: 10.1074/jbc.274.26.18503 10373459

[90] Sledjeski D, Gottesman S. A small RNA acts as an antisilencer of the H-NS-silenced *rcsA* gene of *Escherichia coli*. Proc Natl Acad Sci U S A. 1995;92(6):2003–7. doi: 10.1073/pnas.92.6.2003 7534408 PMC42411

[91] Rozanov DV, D’Ari R, Sineoky SP. RecA-independent pathways of lambdoid prophage induction in *Escherichia coli*. J Bacteriol. 1998;180(23):6306–15. doi: 10.1128/JB.180.23.6306-6315.1998 9829941 PMC107717

[92] Solano C, García B, Valle J, Berasain C, Ghigo J-M, Gamazo C, et al. Genetic analysis of *Salmonella enteritidis* biofilm formation: critical role of cellulose. Mol Microbiol. 2002;43(3):793–808. doi: 10.1046/j.1365-2958.2002.02802.x 11929533

[93] Anriany Y, Sahu SN, Wessels KR, McCann LM, Joseph SW. Alteration of the rugose phenotype in *waaG* and *ddhC* mutants of *Salmonella enterica* serovar Typhimurium DT104 is associated with inverse production of curli and cellulose. Appl Environ Microbiol. 2006;72(7):5002–12. doi: 10.1128/AEM.02868-05 16820499 PMC1489332

[94] García B, Latasa C, Solano C, García-del Portillo F, Gamazo C, Lasa I. Role of the GGDEF protein family in *Salmonella* cellulose biosynthesis and biofilm formation. Mol Microbiol. 2004;54(1):264–77. doi: 10.1111/j.1365-2958.2004.04269.x 15458421

[95] Brown P, Dozois C, Nickerson C, Zuppardo A, Terlonge J, Curtiss R, et al. MlrA, a novel regulator of curli (AgF) and extracellular matrix synthesis by *Escherichia coli* and *Salmonella enterica* serovar Typhimurium. Mol Microbiol. 2001;41(2):349–63. 11489123 10.1046/j.1365-2958.2001.02529.x

[96] Lane M, Simms A, Mobley H. Complex interplay between type 1 fimbrial expression and flagellum-mediated motility of uropathogenic *Escherichia coli*. J Bacteriol. 2007;189(15):5523–33. 17513470 10.1128/JB.00434-07 PMC1951814

[97] Cullen TW, Madsen JA, Ivanov PL, Brodbelt JS, Trent MS. Characterization of unique modification of flagellar rod protein FlgG by *Campylobacter jejuni* lipid A phosphoethanolamine transferase, linking bacterial locomotion and antimicrobial peptide resistance. J Biol Chem. 2012;287(5):3326–36. doi: 10.1074/jbc.M111.321737 22158617 PMC3270987

[98] Cullen T, Trent M. A link between the assembly of flagella and lipooligosaccharide of the Gram-negative bacterium. Proc Natl Acad Sci U S A. 2010;107(11):5160–5. 20194750 10.1073/pnas.0913451107 PMC2841920

[99] Sievers F, Wilm A, Dineen D, Gibson TJ, Karplus K, Li W, et al. Fast, scalable generation of high-quality protein multiple sequence alignments using Clustal Omega. Mol Syst Biol. 2011;7:539. doi: 10.1038/msb.2011.75 21988835 PMC3261699

[100] Waterhouse AM, Procter JB, Martin DMA, Clamp M, Barton GJ. Jalview Version 2--a multiple sequence alignment editor and analysis workbench. Bioinformatics. 2009;25(9):1189–91. doi: 10.1093/bioinformatics/btp033 19151095 PMC2672624

