# Supplementary material for: The dual functions of the GTPase BipA in ribosome assembly and surface structure biogenesis in Salmonella enterica serovar Typhimurium
Source: PLoS Pathog. 2025 Apr 9;21(4):e1013047. doi: 10.1371/journal.ppat.1013047 (PMC12013901; doi:10.1371/journal.ppat.1013047)
Supplement: S1 Supporting Information — This section provides detailed descriptions of the experimental procedures, including cell morphology observation, the swarming motility assay, the deflagellation method, growth curve measurement, the Epsilometer test, and the ATP assay. (DOCX) [file ppat.1013047.s001.docx]

**S1 Supporting Information. Supplementary Materials and Methods**

**Observation of cell morphology**

To observe capsule formation, overnight cultures were diluted to an OD_600_ of 0.04 and further diluted 10^−3^-fold. A volume of 100 μL from the final dilution was spread on an LB agar plate using glass beads. Images of the small colonies were obtained using the Azure C200 Gel Imaging System (Azure Biosystems). For the macrocolony assay, overnight cultures were diluted to an OD_600_ of 0.02, and 3 μL of the diluted cultures were spotted on MacConkey agar plates. The plates were incubated at 37°C or 20°C, and the macrocolonies were photographed using a Canon EOS 5D Mark IV camera.

**Swarming motility assays**

The cultures with an OD_600_ of 0.5, incubated at 37°C, were concentrated 10-fold by centrifugation, and 5 μL of the suspension was spotted onto an LB plate containing 0.6% Bacto agar and 0.5% glucose. The LB agar medium was cooled to 50°C, poured into 25-mL portions, and dried at room temperature for 3 h before use. Incubation commenced 10 min after spotting, and the plates were photographed using the Azure C200 Gel Imaging System (Azure Biosystems).

**Deflagellation methods**

Cells in the exponential phase, grown in LB medium, were collected by centrifugation at 3,000 rpm to minimize flagella loss. The cell pellet was resuspended in 1 mL of PBS, and the flagella were separated by vortexing for 30 min. After centrifugation at 13,000 rpm for 2 min, 900 μL of the supernatant was transferred to a new tube and mixed with 100 μL of 100% trichloroacetic acid. The mixture was incubated at 4°C for 1 h. After centrifugation, the supernatant was removed, and the pellet was washed twice with 200 μL of ice-cold 100% acetone. The resulting pellet was dried and resuspended in 1X SDS dye.

**Growth curve measure under stress conditions**

The overnight cultures were diluted 200-fold in LB medium. A 180 μL aliquot of the diluted culture was dispensed into each well of a black 96-well plate with a clear bottom. Then, 20 μL of serially diluted polymyxin B, H_2_O_2_, or distilled water was added to achieve the desired final concentration. The plates were incubated in a BioTek Epoch2 microplate spectrophotometer (Agilent Technologies) at 37°C for 18 h or 20°C for 48 h with shaking, during which OD_600_ was measured at regular intervals.

**Epsilometer test (E-test)**

The overnight cultures were diluted to an OD_600_ of 0.04 in LB medium and further diluted 10^-2^-fold. A 0.9 mL aliquot of the final dilution was spread onto LB agar plates and allowed to dry for 20 min. For the E-test, a polymyxin B MIC test strip (0.064-1,024 μg/mL, Liofilchem) was placed at the center of plate. The plates were incubated at 37°C or 20°C.

**Intracellular ATP assay**

Cells were grown at 37°C or 20°C to the early exponential phase in LB medium and adjusted to an OD_600_ of 0.5. A 10 μL aliquot of the culture was diluted into 90 μL of preheated dilution buffer (100 mM Tris-HCl [pH 8.0], 4 mM EDTA) and boiled for 2 min. The samples were then centrifugated at 13,000 rpm for 1 min, and the supernatant was transferred to a new tube and stored at -80°C until luminescence measurement. A 50 μL aliquot of the supernatant was dispensed into a black 96-well plate, followed by the addition of 50 μL of luciferase reagent (ATP Bioluminescence Assay Kit CLS II, Roche). Luminescence was measured and integrated over 1 to 10 s using a BioTek Synergy H1 Plate Reader (Agilent Technologies). To quantify intracellular ATP levels, an ATP standard curve was generated using known concentrations of ATP provided in the kit, and sample ATP concentrations were calculated based on the standard curve.
