# Supplementary figures and images for: The dual functions of the GTPase BipA in ribosome assembly and surface structure biogenesis in Salmonella enterica serovar Typhimurium
Source: PLoS Pathog. 2025 Apr 9;21(4):e1013047. doi: 10.1371/journal.ppat.1013047 (PMC12013901; doi:10.1371/journal.ppat.1013047)

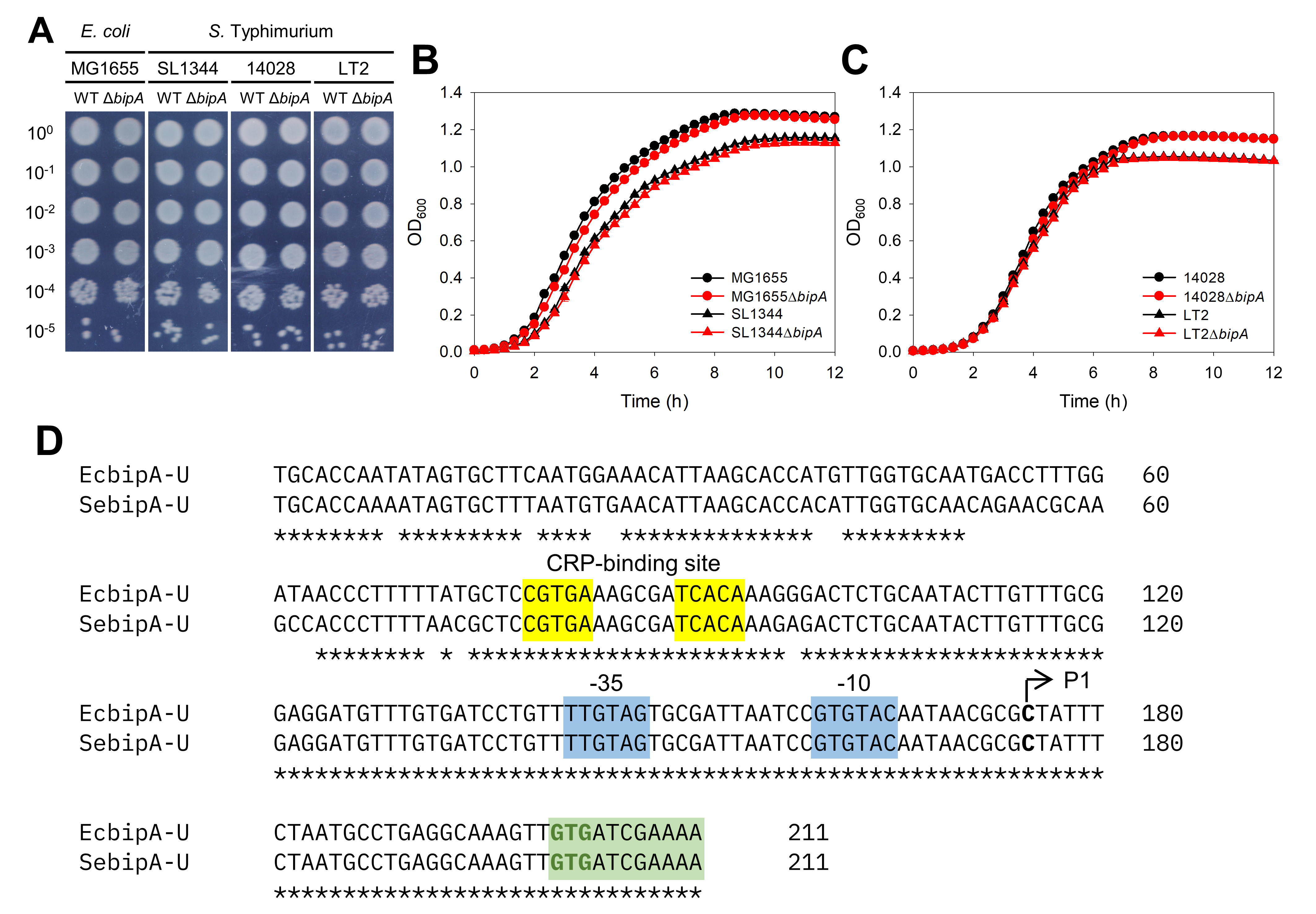

Supplement: S1 Fig — The growth of wild-type and bipA-deleted strains of E. coli and S. Typhimurium was assessed at 37°C. (A) Overnight cultures were diluted and spotted, as described in Fig 1A, followed by incubation at 37°C. (B and C) Cells grown at 37°C to an OD600 of 0.5 were inoculated into fresh LB medium and further incubated at 37°C for 12 h. Error bars indicate SD. (D) Conserved CRP-binding site upstream of the SebipA promoter. The upstream DNA sequences of E. coli and S. Typhimurium are aligned, with the consensus CRP-binding site highlighted in yellow, the −35 and −10 regions in blue, and the initiation codon GTG in green. (TIF) [file ppat.1013047.s002.tif]

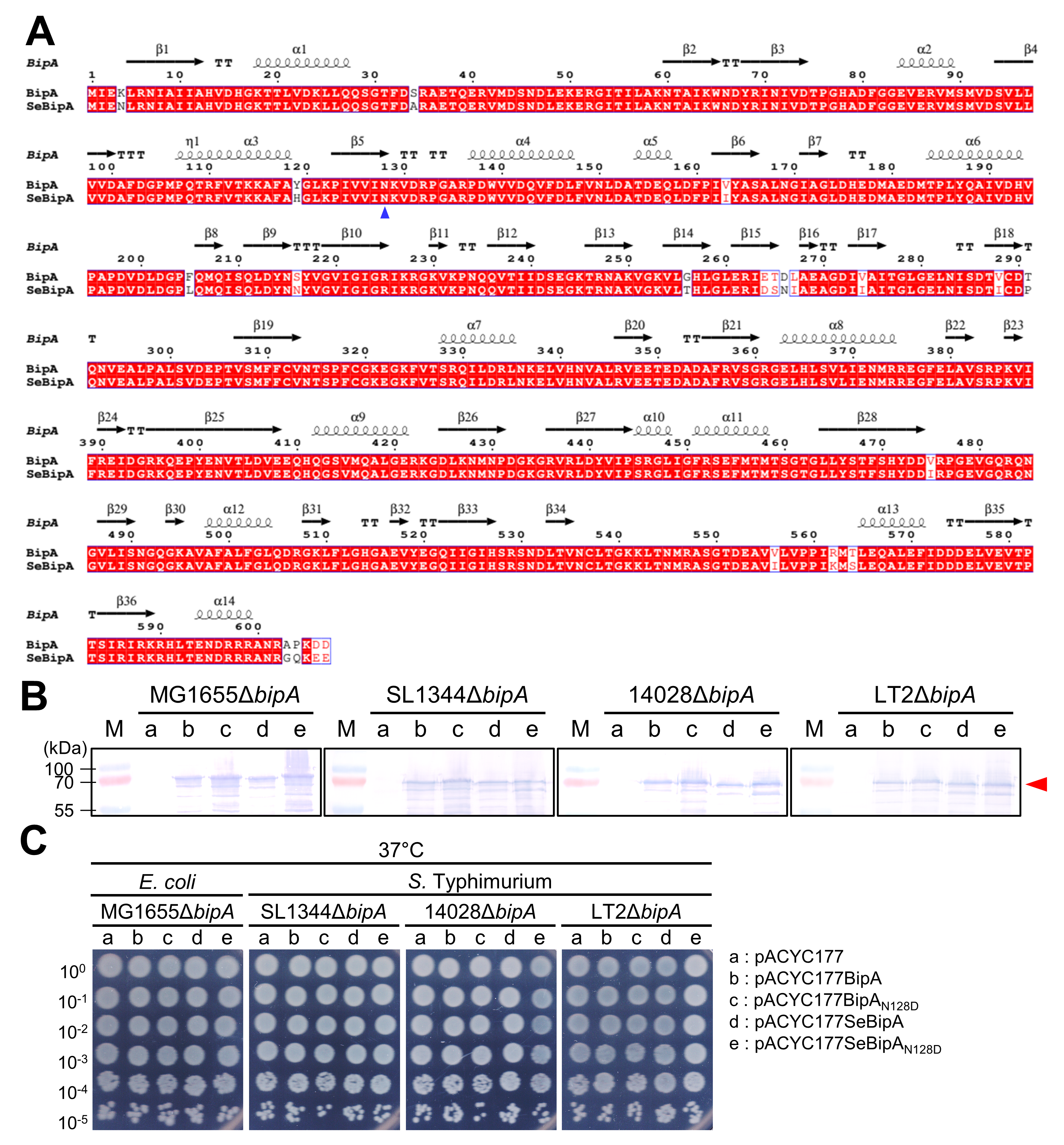

Supplement: S2 Fig — (A) Sequence alignment of BipA from E. coli and S. Typhimurium was conducted using ClustalW and ESPript. The numbers indicate the corresponding residues, and the blue arrowhead indicates the Asn128 residue. (BipA, GenBank accession number: AAT48232; SeBipA, CAC14270). (B) Expression of wild-type and mutant BipA proteins in MG1655ΔbipA and mutant S. Typhimurium cells. The transformants shown in Fig 2B underwent Western blot analysis using an anti-BipA antibody. M: PageRuler Prestained Protein Ladder (Thermo Fisher Scientific). (C) Colony formation of cross-complemented bipA-deletion strains at 37°C. The bipA-deleted E. coli and S. Typhimurium strains were transformed with the plasmid as shown. Transformants were spotted as described in Fig 2B, and the plates were incubated at 37°C. (TIF) [file ppat.1013047.s003.tif]

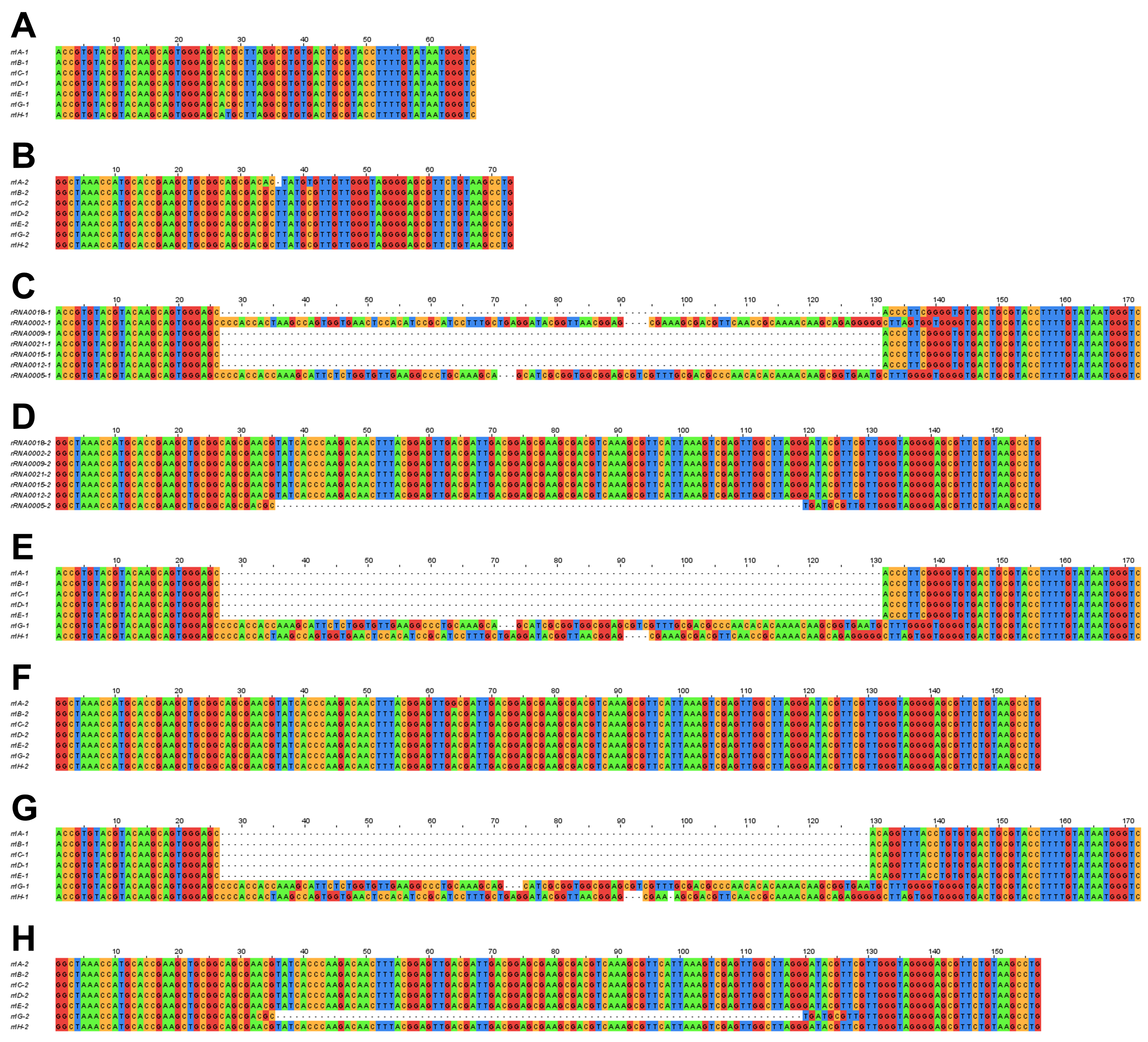

Supplement: S3 Fig — The nucleotide sequences of the 23S rRNA genes from E. coli MG1655 (A and B) and S. Typhimurium SL1344 (C and D), 14028 (E and F), and LT2 (G and H) were analyzed. The first IVSs in the helix 25 region (A, C, E, and G) and the second IVSs in the helix 45 region (B, D, F, and H) were aligned using Clustal Omega [99] and visualized using Jalview [100]. (TIF) [file ppat.1013047.s004.tif]

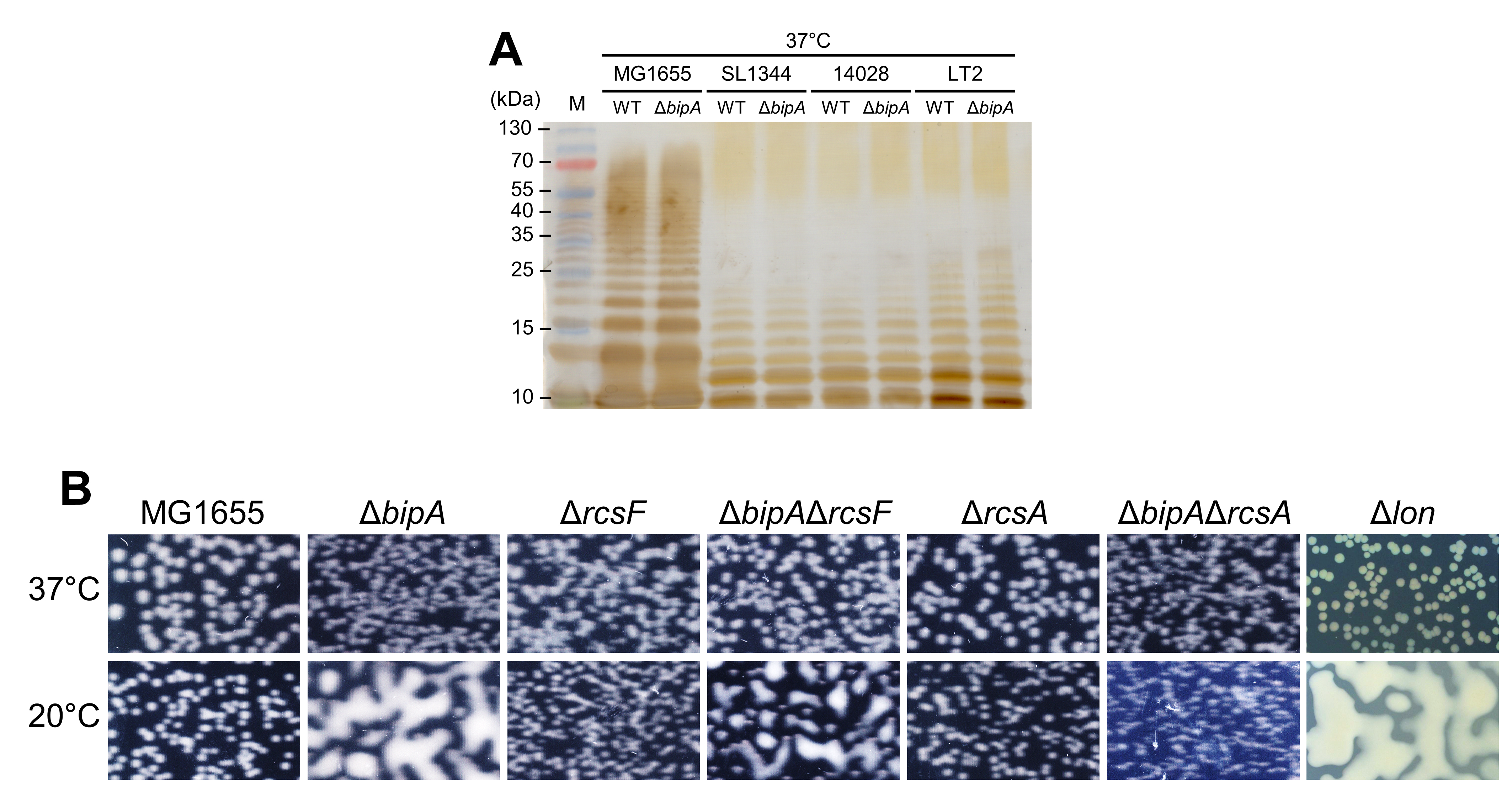

Supplement: S4 Fig — (A) LPS profile analysis of the E. coli and S. Typhimurium strains. LPS was extracted and analyzed, as described in Fig 5. (B) Colony morphology of bipA, rcsF, rcsA, and lon mutants. Overnight cultures were diluted to an OD600 of 0.04 in the LB medium and then further diluted 10−3-fold. One hundred microliters of the diluted culture were spread with glass beads on LB agar plates and incubated at 37°C or 20°C. (TIF) [file ppat.1013047.s005.tif]

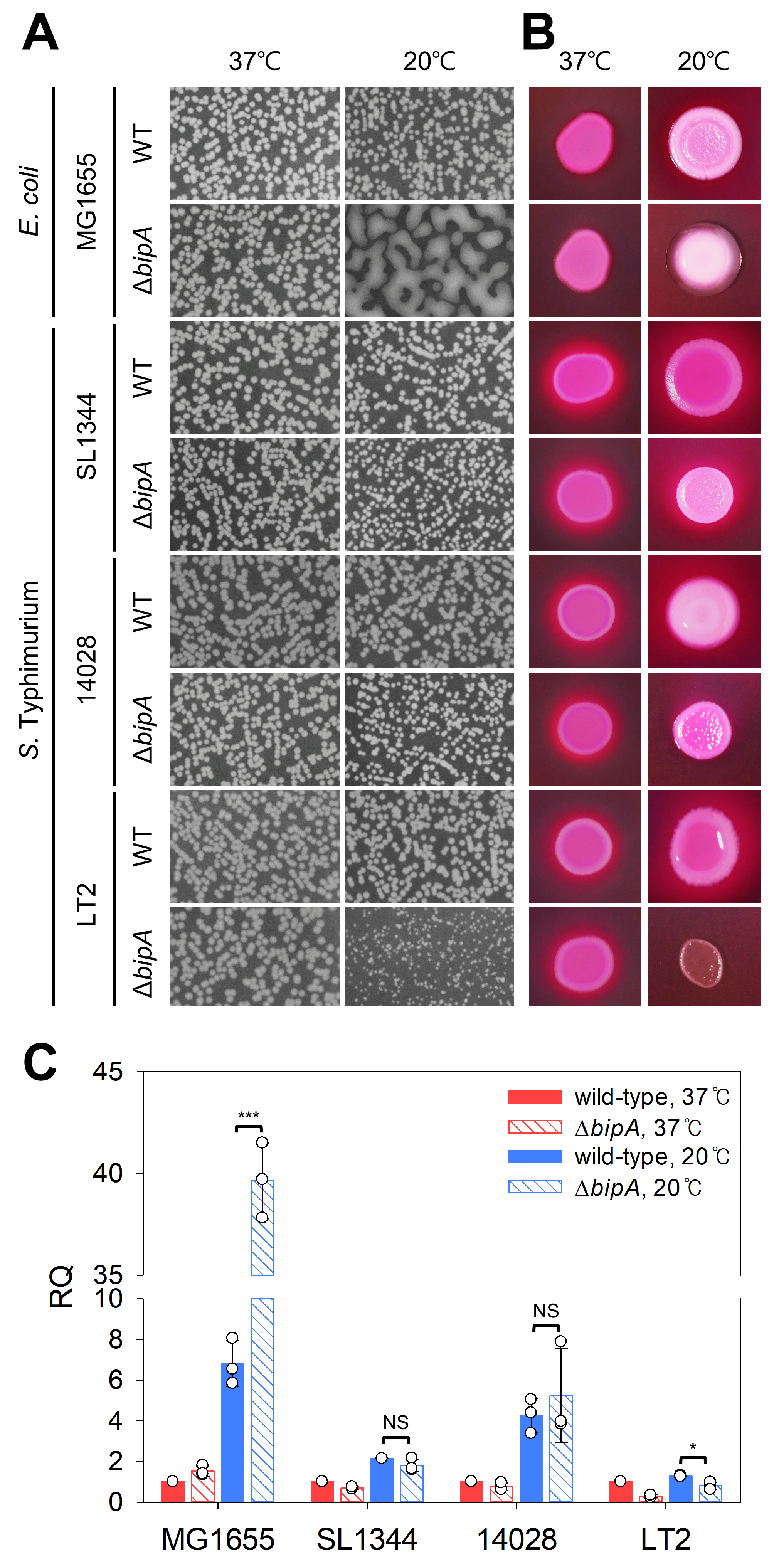

Supplement: S5 Fig — (A) Colony morphology of wild-type and bipA-deleted strains of E. coli and S. Typhimurium. Overnight cultures were diluted and spread as in S4B Fig. (B) Macrocolony assay of the same strains at 37°C for 18 h or 20°C for 7 d. Overnight cultures were diluted to an OD600 of 0.02 in the same medium, and 3 μL of the diluted cultures were spotted on MacConkey agar plates. (C) Relative quantification of gmd mRNA levels in the cps cluster. Total RNA was extracted from cells grown to an OD600 of 0.5 and analyzed using qRT-PCR. The relative quantity was normalized to the rrsA gene and expressed relative to the transcript level of the wild-type strain grown at 37°C. (TIF) [file ppat.1013047.s006.tif]

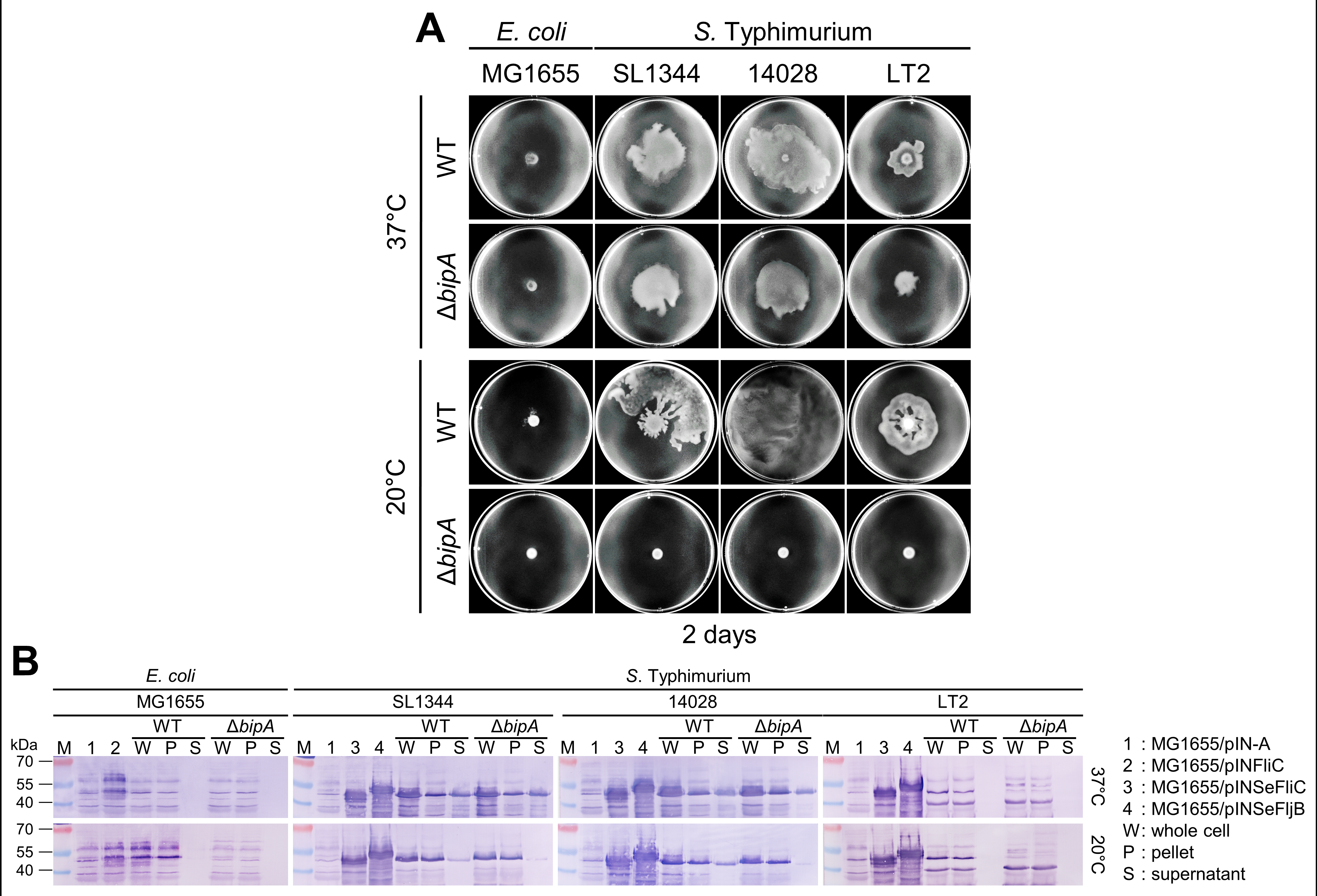

Supplement: S6 Fig — (A) Swarming motility assay of wild-type and bipA-deleted strains of E. coli and S. Typhimurium at 37°C or 20°C. The cells were grown and diluted, as described in Fig 7A. The cultures were concentrated 10-fold and spotted on an LB agar plate containing glucose. Plates were incubated at 37°C for 14 h or 20°C for 2 d. (B) Reduced flagellin production in the ΔbipA of S. Typhimurium at 20°C. Overnight cultures were diluted 10−2-fold in fresh medium and incubated until the OD600 reached 0.5 at 37°C or 20°C. Deflagellation was performed as described in the Materials and Methods section. Western blot analysis of the wild-type and bipA-deleted strain samples was conducted using an anti-FliC antibody. To determine the locations of FliC and FljB, the lysates of MG1655 cells harboring pIN-A, pINFliC, pINSeFliC, or pINSeFljB were loaded onto SDS-PAGE. (TIF) [file ppat.1013047.s007.tif]

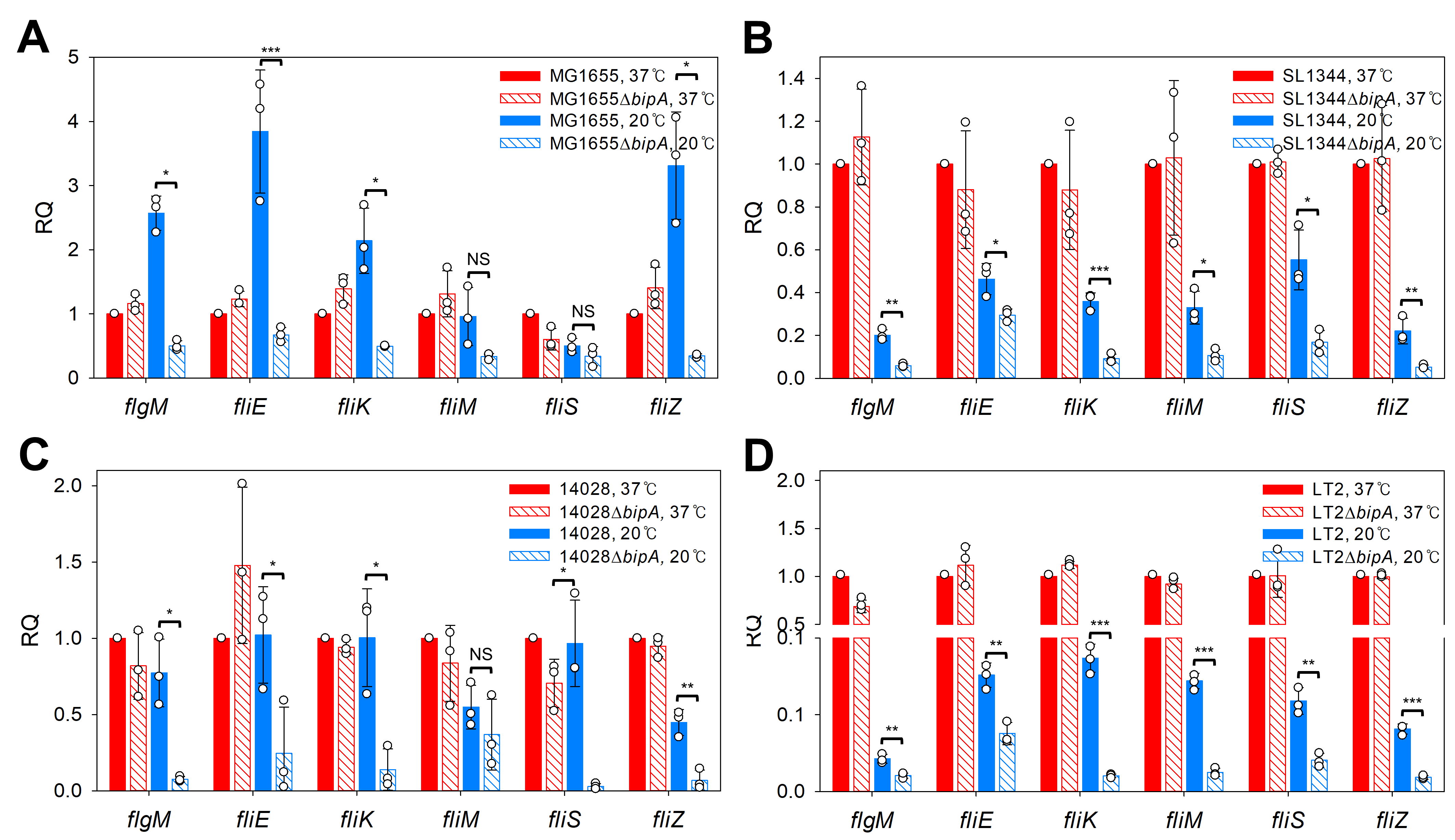

Supplement: S7 Fig — Total RNA was extracted from the wild-type and bipA-deleted strains cultured in the early exponential phase at 37°C or 20°C and analyzed using qRT-PCR. The relative expression levels of flgM, fliE, fliK, fliM, fliS, and fliZ were normalized to the endogenous control gene rrsA. (TIF) [file ppat.1013047.s008.tif]

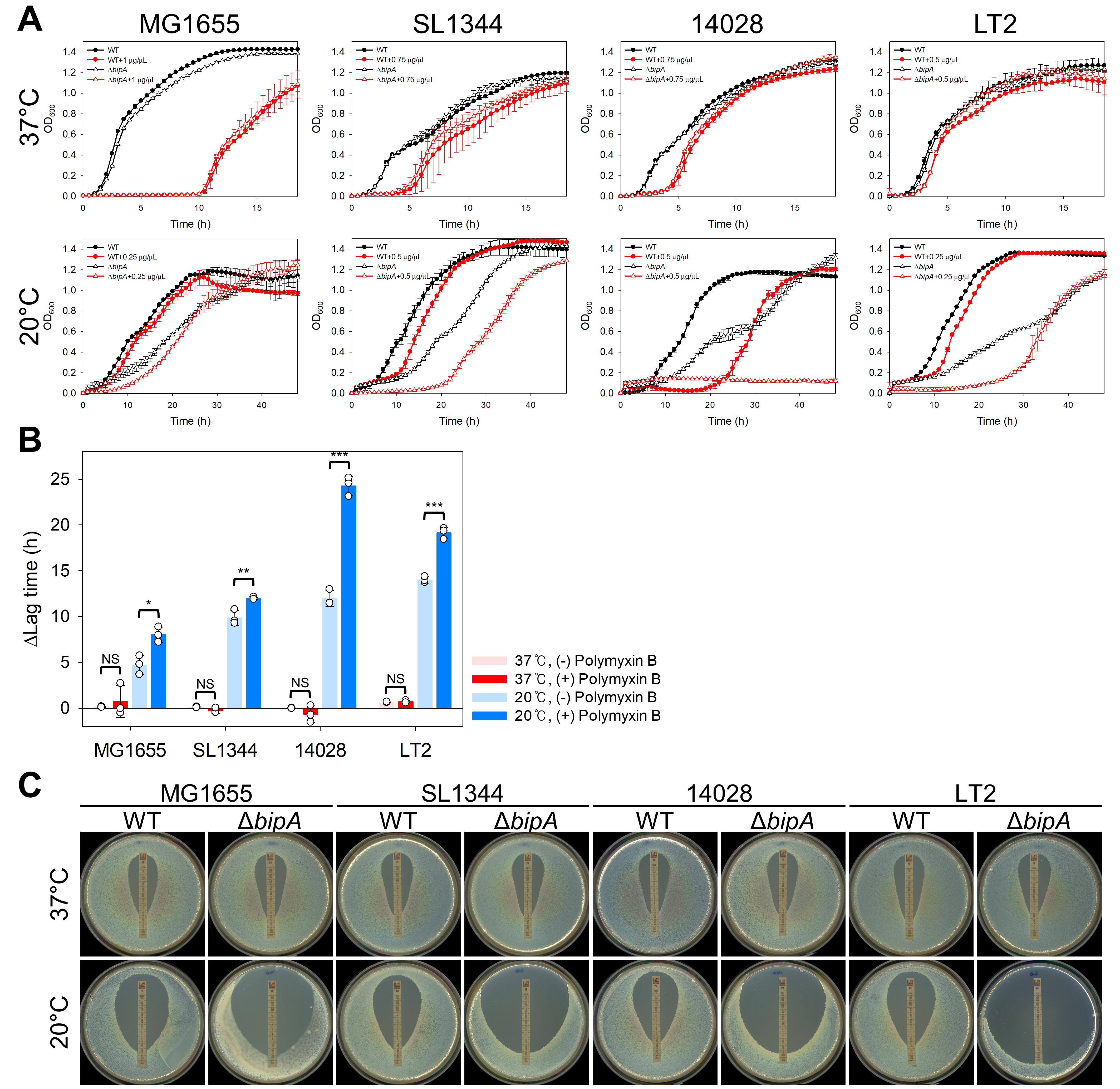

Supplement: S8 Fig — (A) Growth curves of wild-type and bipA-deleted strains in the presence of polymyxin B. Overnight cultures were diluted 200-fold in fresh LB medium. A 180 μL aliquot of the diluted culture was added to each well of a 96-well plate, along with 20 μL of polymyxin B or distilled water as a control. Plates were incubated at 37°C for 18 h or at 20°C for 48 h with shaking. (B) ΔLag time between wild-type and bipA-deleted strains. Lag time was determined using Gen5 software (Agilent Technologies). ΔLag time was calculated as the lag time of the mutant strain minus the lag time of the wild-type strain. (C) Polymyxin B E-test for MIC determination. A polymyxin B MIC test strip was placed onto LB agar plates spread with diluted cultures of wild-type or bipA-deleted strains, followed by incubation at 37°C or 20°C. (TIF) [file ppat.1013047.s009.tif]

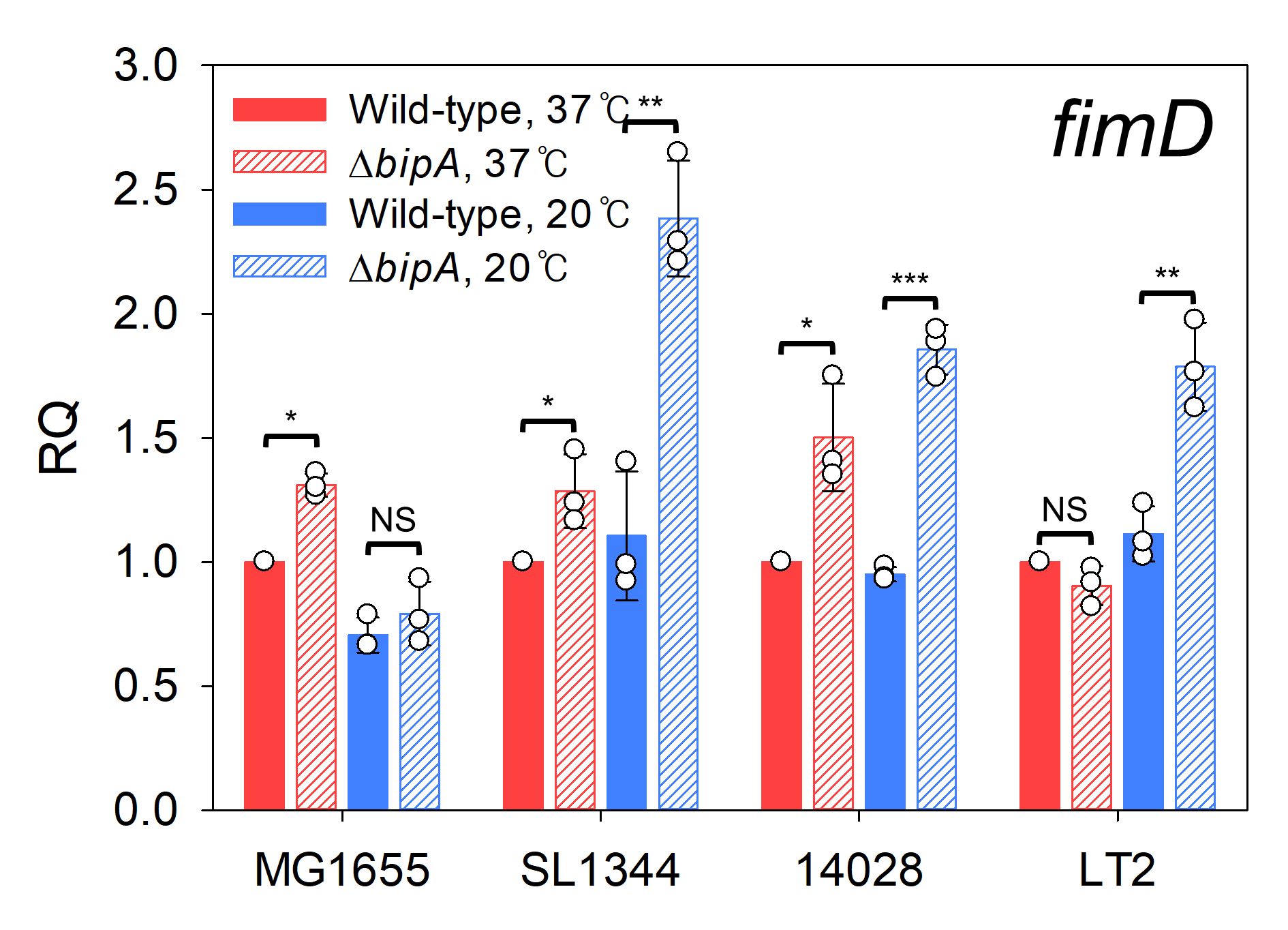

Supplement: S9 Fig — Total RNA was extracted from wild-type and bipA-deleted strains in the early exponential phase at 37°C or 20°C and analyzed using qRT-PCR. The relative expression levels of the type 1 fimbriae gene fimD were normalized to the endogenous control gene rrsA. (TIF) [file ppat.1013047.s010.tif]

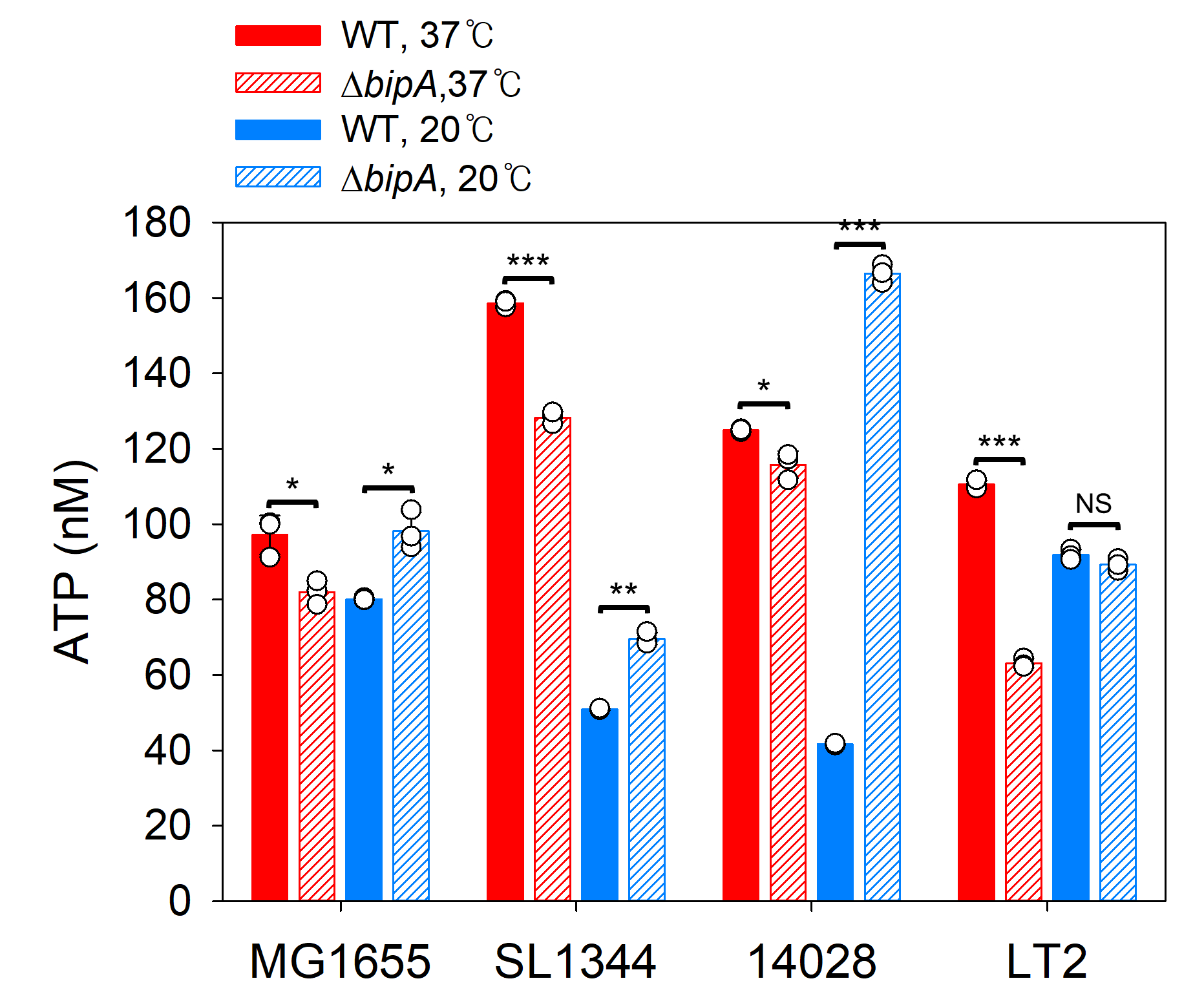

Supplement: S10 Fig — Lysate samples were prepared from wild-type and bipA-deleted strains cultured in the early exponential phase at 37°C or 20°C, as described in S1 Supporting Information. A 50 μL sample was mixed with 50 μL of luciferase reagent, followed by luminescence measurement. ATP concentrations were calculated based on a standard curve ranging from 10-5-10-10 M. (TIF) [file ppat.1013047.s011.tif]

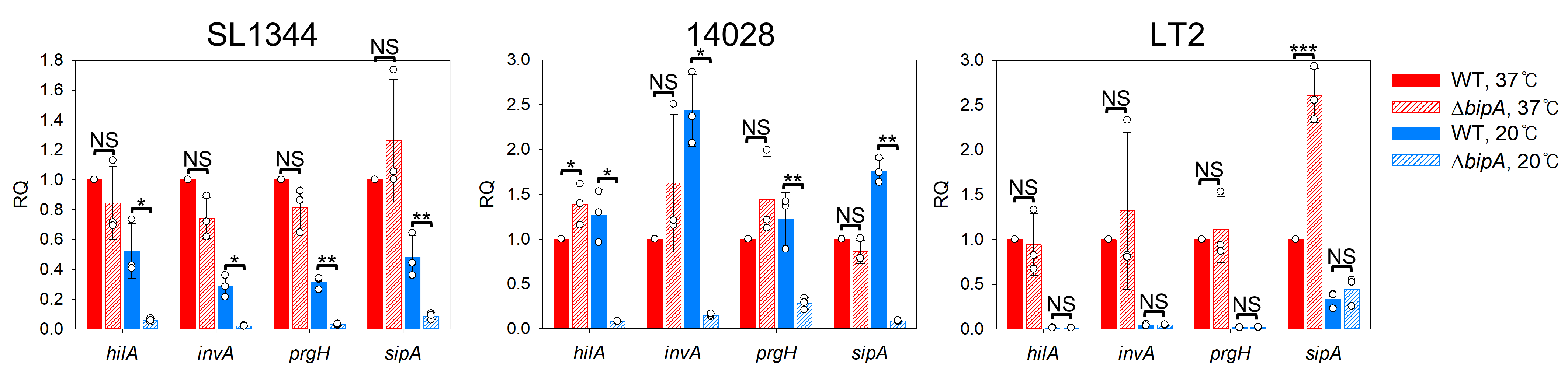

Supplement: S11 Fig — Total RNA was extracted from the wild-type and bipA-deleted cells incubated at 37°C or 20°C to the early exponential phase and analyzed using qRT-PCR. The relative expression levels of hilA, invA, prgH, and sipA were normalized to the endogenous control gene rrsA. (TIF) [file ppat.1013047.s012.tif]

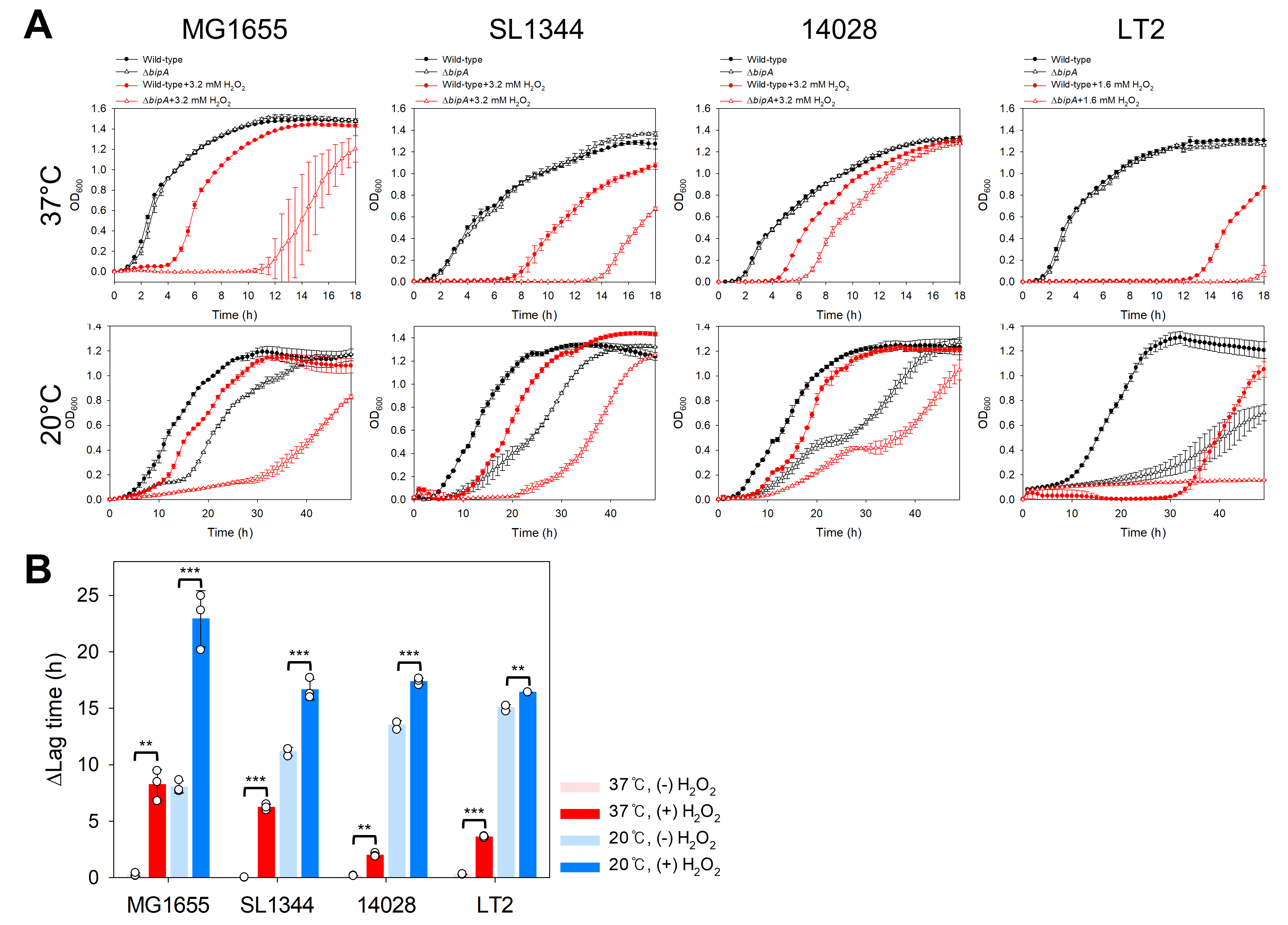

Supplement: S12 Fig — (A) Growth curves of wild-type and bipA-deleted strains of E. coli and S. Typhimurium in the presence of H2O2. Growth was measured as described in S8A Fig. (B) Increased lag time in bipA-deleted strains in the presence of H2O2. ΔLag time was calculated as described in S8B Fig. (TIF) [file ppat.1013047.s013.tif]
