# Supplementary material for: The dual functions of the GTPase BipA in ribosome assembly and surface structure biogenesis in Salmonella enterica serovar Typhimurium
Source: PLoS Pathog. 2025 Apr 9;21(4):e1013047. doi: 10.1371/journal.ppat.1013047 (PMC12013901; doi:10.1371/journal.ppat.1013047)
Supplement: S1 Table — List of primers used for strains and plasmids construction, and qRT-PCR. (DOCX) [file ppat.1013047.s014.docx]

**S1 Table. Primers used in this study**

| **Primers** | **Sequence (5'→3')** | **References** |
| --- | --- | --- |
| **Strain construction** | | |
| SebipA-del-F | TGTACAATAACGCGCTATTTCTAATGCCTGAGGCAAAGTTGTGTAGGCTGGAGCTGCTTC | This study |
| SebipA-del-R | GCTTTCATTTCGGCAGGGTTTTTATCCGTAAGAGCGTTAAATTCCGGGGATCCGTCGACC | This study |
| **Plasmid construction** | | |
| bipA-100bp-U | CTCTGCAATACTTGTTTG | [27] |
| bipA-100bp-D | CCACAGACTTATAAGGGAG | [27] |
| SebipA-200-U | CACGGTTATGTTGCACC | This study |
| SebipA-200-D | GCTACGTTTAGCCGGATTGG | This study |
| SebipA-5-Ndel | GGCATATGATCGAAAATTTGCGTAACATC | This study |
| SebipA-3-Hindlll | ATAAGCTTTTACTCTTCTTTCTGACCACG | This study |
| EcfliC-F | GACATATGGCACAAGTCATTAATACCAAC | This study |
| EcfliC-R | ATAAGCTTTTAACCCTGCAGCAGAGACA | This study |
| SefliC-F | GCCATATGGCACAAGTCATTAATACAAACA | This study |
| SefliC-R | ACAAGCTTTTAACGCAGTAAAGAGAGGAC | This study |
| SefljB-F | GGCATATGGCACAAGTAATCAACACTAAC | This study |
| SefljB-R | CTAAGCTTTTAACGTAACAGAGACAGCACG | This study |
| SeBipA-N128D-F | GAAACCCATTGTGGTTATCGACAAAGTTGACCGTCCT | This study |
| SeBipA-N128D-R | AGGACGGTCAACTTTGTCGATAACCACAATGGGTTTC | This study |
| pagP-200-U | CATCGCAGAAAACGACGCATC | This study |
| papP-200-D | GACACAAATGCTGTGTCGGTTAC | This study |
| **qRT-PCR** | | |
| EcflgM-RT-F | GAAGCCTGTAAGCACCGT | This study |
| EcflgM-RT-R | TTGATCAGCGCATCGGCAA | This study |
| SeflgM-RT-F | CTTTGAAACCCGTTAGCAC | This study |
| SeflgM-RT-R | GAGCGAGTCTGCTATTTTTC | This study |
| fliE-RT-F | CAGGGGATTGAAGGGGTT | This study |
| fliE-RT-R | CGCACCTGAATCCCCATT | This study |
| EcfliK-RT-F | CAGAGTAAAGCGGAAGTCA | This study |
| EcfliK-RT-R | CATCCACTTTGAGGGAGATT | This study |
| SefliK-RT-F | GCATCTACTGTCTACGCTAA | This study |
| SefliK-RT-R | CCTTTTTCATGCTCGCTAAA | This study |
| fliM-RT-F | CCGACCAACCTGAACCT | This study |
| fliM-RT-R | GTGATATTGGTAAATTTCACCTGC | This study |
| EcfliS-RT-F | ATGCTATTTGATGGAGTGCT | This study |
| EcfliS-RT-R | TCGTCTTTGCTCTCTTCATC | This study |
| SefliS-RT-F | ATCTTTCCGAGCTATACGAC | This study |
| SefliS-RT-R | TTAACGAGACTCCTGGAAAG | This study |
| EcfliZ-RT-F | AACGGAACAAAAATCATGGG | This study |
| EcfliZ-RT-R | CTCGTGTAGATGATTCCCC | This study |
| SefliZ-RT-F | ATCTTAAAGACTTTAAACACAGCCA | This study |
| SefliZ-RT-R | CCGATAATATCGAAAAAATCACTCT | This study |
| gmd-RT-F | TTGAAGAGAAGGGCATTGTG | [28] |
| gmd-RT-R | GAGATTTCAGCAGAGAGTGT | [28] |
| Segmd-RT-F | TCAAAAGTCGCTCTCATTACTG | This study |
| Segmd-RT-R | CCAGGTTGTAGACCTCATC | This study |
| SehilA-RT-F | AAAGAATATGCCGTTCTGGT | This study |
| SehilA-RT-R | ATGTATGAGTCGTAGGTTGC | This study |
| SeinvA-RT-F | TATTGTTACGAGATGGCGAG | This study |
| SeinvA-RT-R | CTACCTTGCTGATGGATTGT | This study |
| SeprgH-RT-F | AGAGAAGACGATAACAAGCC | This study |
| SeprgH-RT-R | GCAGGTATATCAGGGAGTTG | This study |
| SesipA-RT-F | CAACCATGATAATAGCCGGA | This study |
| SesipA-RT-R | CTTTACTTTCGGATGAAGCG | This study |
| fimD-RT-F | TACCAATACCCTGGCTGATA | This study |
| fimD-RT-R | TGAGCATTTTCCTCTTCTCC | This study |
| SefimD-RT-F | AAGTTATTCGCTGTCATGGT | This study |
| SefimD-RT-R | CTCAGATTACGTCCTTCCAG | This study |
